# Global burden of type 1 diabetes mellitus in women of childbearing age from 1990 to 2021 with projections to 2030

**DOI:** 10.1097/MD.0000000000044419

**Published:** 2025-09-19

**Authors:** Zhenhao Liu, Kui Wang, Guangzhong Zeng, Wei Pan, Ling Zhu

**Affiliations:** a Department of Cardiovascular Medicine, Ping Xiang People’s Hospital, Jiangxi Province, China; b The First Clinical Medical College, Qilu Hospital of Shandong University, Jinan, Shandong Province, China; c Department of Cardiology, Department of Geriatrics, Foshan Women and Children Hospital, Foshan, Guangdong Province, China; d General Medicine Department, The First People’s Hospital of Jingdezhen, Jingdezhen, Jiangxi Province, China.

**Keywords:** epidemiology, Global Burden of Disease, risk factors, type 1 diabetes mellitus, women of childbearing age

## Abstract

Type 1 diabetes mellitus (T1DM) presents significant challenge for women of childbearing age (WCBA), yet current data on its burden are lacking. This study examines the global, regional, and national burden of T1DM in WCBA from 1990 to 2021 and projects trends to 2030. Data from the Global Burden of Disease 2021 were analyzed for T1DM among WCBA in 204 countries and territories since 1990. We analyzed prevalence, disability-adjusted life years (DALYs), and mortality rates, along with DALYs attributable to risk factors. The Bayesian age-period-cohort model was employed for projections through 2030. From 1990 to 2021, global T1DM prevalent cases in WCBA increased by 73%, with a 15% increase in the age-standardized prevalence rate and an average annual percentage change (AAPC) of 0.45%. DALYs increased by 33%, though the age-standardized DALYs rate decreased from 53.16 to 47.11 per 100,000 (AAPC −0.48%). Mortality increase by 19%, yet the age-standardized mortality rate decreased from 0.70 to 0.54 per 100,000 (AAPC −0.92%). Prevalence and DALYs mainly increased in older age groups, while mortality decreased across all age groups. Projections indicate continued prevalence growth, with further decreases in DALYs and mortality. Substantial differences exist across various regions and countries globally. Significant regional and country disparities were observed, particularly in low sociodemographic index regions, with high fasting plasma glucose and temperature extremes as key risk factors. Despite increasing T1DM prevalence, improved disease management and public health initiatives have led to decreases in DALYs and mortality rates. Enhanced diabetes education, care access, and effective management strategies are essential for reducing the global T1DM burden among WCBA.

## 1. Introduction

Diabetes is a group of metabolic disorders characterized by hyperglycemia and classified into several types, including type 1 diabetes mellitus (T1DM), type 2 diabetes mellitus, and gestational diabetes based on their etiology.^[1]^ T1DM, also known as insulin-dependent diabetes, is an autoimmune response against pancreatic beta-cell proteins and has developed into a major global health concern.^[2]^ Recent estimates place its global prevalence at approximately 8.4 million in 2021 and projecting an increase to 17.4 million by 2040.^[3]^ Although T1DM is traditionally considered a juvenile autoimmune disease, it is increasingly gaining attention in women of childbearing age (WCBA).

WCBA with T1DM may face increased health risks, including gestational hyperglycemia, pregnancy-induced hypertension, preeclampsia, and diabetic ketoacidosis.^[4]^ These complications not only affect the health of WCBA but can also have severe adverse effects on fetal development, such as preterm birth, stillbirth, congenital anomalies, and perinatal mortality.^[5]^ Moreover, such conditions can also impact the long-term health of the mother, potentially increasing the risk of cardiovascular diseases and other metabolic disorders.^[6]^ Furthermore, the management of T1DM during pregnancy poses substantial challenges, requiring strict monitoring of blood glucose levels and careful adjustments to insulin therapy to prevent pregnancy-related complications.^[7]^

Previous studies on global burden of T1DM have primarily focused on adolescents and young adults.^[8]^ Although the impact of T1DM in WCBA is crucial for public health issue and has been subject to extensive research, most existing research focuses on pregnancy outcomes and short-term complications. Due to methodological heterogeneity, there has been inadequate exploration of the variations across different countries or regions, leading to an absence of a comprehensive global perspective. To provide further epidemiological evidence, elucidate advancements in disease management, and assist countries and territories in developing targeted strategies for prevention and control, a thorough and detailed analysis of the T1DM burden is essential.

In response to these gaps, we utilized data from the 2021 Global Burden of Disease, Injuries, and Risk Factors Study (GBD) to report the prevalence, disability-adjusted life years (DALYs), and mortality rates associated with T1DM from 1990 to 2021 globally, regionally, and nationally, along with attributed risk factors, stratified by age group and the sociodemographic index (SDI). Additionally, we conducted predictive analyses to estimate the burden of T1DM in WCBA worldwide for the year 2030.

## 2. Methods

### 2.1. Data acquisition and download

The data for this study were obtained from the GBD 2021 dataset, which provides comprehensive information on the global and regional burden of 371 diseases, injuries, and impairments, as well as 88 risk factors, encompassing 204 countries and territories from 1990 to 2021, utilizing the most recent epidemiological data and enhanced standardized methodologies.^[9]^ Supported by over 11,500 collaborators from 164 countries, the GBD 2021 systematically evaluates global health and disease burden through extensive data collection, review, and analysis. Specifically, data on prevalence, DALYs, and mortality of T1DM in WCBA, as well as age, location, and rates, and DALYs attributable to each risk factor were accessed and downloaded via the Global Health Data Exchange platform (https://vizhub.healthdata.org/gbd-results/). SDI data were also obtained to assess the impact of socioeconomic factors on disease burden.

The GBD 2021 also produced a SDI for each country or territory online (https://ghdx.healthdata.org/gbd-2021), providing a comprehensive measure of income, education, and fertility conditions. The SDI values ranged from 0 to 1, with higher values indicating greater socioeconomic development levels. Based on the 2021 SDI values, countries were grouped into 5 quintiles: high, high-middle, middle, low-middle, and low.

### 2.2. Data sources and disease model

In the GBD 2021 study, the reference case definition for diabetes was a fasting plasma glucose (FPG) concentration of 7 mmol/L (126 mg/dL) or higher, or a person using insulin or diabetes medication. Data sources for T1DM included surveys, population censuses, health statistics, vital registration data, hospital records, diabetes registries, and insurance claims. Specifically, for those under the age of 15, insurance claims provided critical data due to the precision in reflecting T1DM incidence. Most of the inputs came from insurance, registry, and survey data, which were systematically screened via the Global Health Data Exchange for comprehensiveness and reliability.

To estimate T1DM mortality, the Cause of Death Ensemble model (CODEm) was used. CODEm combines data from multiple sources, automatically selecting the best-fitting model through Bayesian inference. Its ability to adjust for variations between data sources and across regions, ages, and genders ensures accurate mortality estimates. Additionally, Meta-Regression-Bayesian, Regularized, Trimmed was used to correct biases between data sources. By identifying overlapping data points between alternative and reference case definitions, Meta-Regression-Bayesian, Regularized, Trimmed converts them into logit-space differences, and applies random effects meta-regression to generate adjusted estimates.

DisMod-MR 2.1 was the primary tool for estimating T1DM prevalence and excess mortality in GBD 2021. The model processed input data from various countries, regions, and time periods, adjusting estimates with covariates like maternal age and education. Age-specific priors, such as a zero incidence for ages 0 to 1 and an upper limit of 0.0006 for ages 1 to 20, ensured accurate global burden estimates. Detailed modeling strategies for T1DM can be found in the GBD 2021 methods appendices (https://www.healthdata.org/gbd/methods-appendices-2021).

### 2.3. Study population and statistics analysis

WCBA denotes the phase of women with reproductive ability and experiences cyclical changes in sex hormones. The WHO defined WCBA as women aged 15 to 49 years.^[10]^ In this study, we extracted data on T1DM in WCBA stratified 7 age groups (15–19, 20–24, 25–29, 30–34, 35–39, 40–44, 45–49 years).

We analyzed the relationship between the burden of T1DM and SDI regions and 204 countries and territories was examined using smoothing splines models. The expected values were calculated considering the SDI and rates across all locations. Locally Weighted Scatterplot Smoothing and Spearman correlation were used to estimate the *R* indices and *P* values for the association between age-standardized rates with SDl.

A comprehensive assessment was conducted to quantify the burden of T1DM, encompassing its prevalence, DALYs, and mortality. Additionally, the investigation delved into the demographic variables influencing T1DM, examining the distribution of the diseases burden across different age groups and SDI. We calculated the age-standardized prevalence rate (ASPR), age-standardized DALYs rate (ASDR), age-standardized mortality rate (ASMR), and their corresponding 95% confidence intervals (CIs) based on the world standard population reported in the 2021 GBD Study for an interregional comparison, and further estimated average annual percentage change (AAPC) from 1990 to 2021.

The AAPC reflects the average rate of change of a specific variable over a given period. In this study, it represents the AAPC calculated from the weighted average of slope coefficients in the joinpoint regression model from 1990 to 2021. The AAPC value indicates whether the variable is increasing, decreasing, or remaining stable each year. If both the annual percentage change estimates and its 95% CIs are >0 or both <0, the corresponding rate is considered to be trending increase or decrease.

To predict the future burden of T1DM in WCBA, we employed the Bayesian age-period-cohort (BAPC) analysis model. The BAPC model, implemented using the INLA and BAPC packages in R, allowed us to forecast the prevalence, DALYs, and mortality of disease through 2030.^[11]^ This model considers the effects of age, period, and cohort, providing a comprehensive approach to understanding future trends in disease burden.^[12]^

In the current study, the prevalence, DALYs, and mortality were represented as projections for per 100,000 population, including of their 95% uncertainty intervals, while AAPCs were shown with their 95% CIs. All statistical analyses and data visualizations were performed using R (version 4.3.3, The R Foundation for Statistical Computing, Vienna, Austria). *P*-values <.05 were considered statistically significant.

### 2.4. Ethics approval

This study does not contain personal or medical information about an identifiable living individual, and animal subjects were not involved in the study.

## 3. Results

### 3.1. Global trends

From 1990 to 2021, the global prevalent cases of T1DM in WCBA increased by 73%, from 2.998 million to 5.18 million. The ASPR increased by 15%, from 228.89 to 263.58 per 100,000 population, with an AAPC of 0.45% (Table 1). During the study period, the ASPR in WCBA was consistently higher than in the overall T1DM population, although the AAPC was lower (Figure S1A, Supplemental Digital Content, https://links.lww.com/MD/Q102; Figure S2A, Supplemental Digital Content, https://links.lww.com/MD/Q102).

**Table 1:** Age-standardized prevalence rate (ASPR) of T1DM in WCBA in 1990 and 2021, and average annual percentage change (AAPC) from 1990 to 2021 at the global and regional level.

	1990		2021		1990–2021	
	Prevalent cases, 000s (95% UI)	ASPRs per 100,000 (95% UI)	Prevalent cases, 000s (95% UI)	ASPRs per 100,000 (95% UI)	Total percent change (95% UI)	AAPC (%) (95% CI)
Global	2998.48 (2435.34–3675.63)	228.89 (186.65–279.59)	5177.16 (4155.5–6429.62)	263.58 (211.25–327.8)	0.73 (0.78 to 0.68)	0.45 (0.44 to 0.46)*
SDI						
High	837.63 (712.12–980.94)	365.28 (310–428.34)	1203.08 (1009.36–1422.83)	479.89 (401.16–569.39)	0.44 (0.38 to 0.50)	0.85 (0.81 to 0.89)
High-middle	510.36 (419.44–618.84)	186.17 (153.27–225.40)	805.62 (644.28–996.04)	253.79 (201.21–315.8)	0.58 (0.50 to 0.66)	1.00 (0.95 to 1.04)
Middle	804.65 (627.55–1025.17)	184.32 (144.74–233.63)	1362.48 (1061.5–1745.39)	217.18 (168.56–279.15)	0.69 (0.61 to 0.80)	0.57 (0.54 to 0.60)
Low-middle	619.90 (487.13–786.82)	232.57 (183.85–293.61)	1252.81 (981.22–1594.55)	249.81 (196.12–317.38)	1.02 (0.97 to 1.08)	0.22 (0.21 to 0.24)
Low	222.45 (180.62–273.45)	204.98 (167.38–250.93)	548.33 (440.42–680.69)	206.21 (166.66–254.75)	1.46 (1.40 to 1.52)	0.02 (−0.03 to 0.07)
Regions						
Andean Latin America	11.35 (8.81–13.98)	124.08 (97.30–151.66)	24.03 (18.92–29.97)	137.47 (108.18–171.54)	1.12 (0.98 to 1.29)	0.38 (0.34 to 0.41)*
Australasia	22.49 (20.91–24.20)	415.96 (386.70–447.89)	46.29 (41.05–52.46)	620.74 (549.16–704.56)	1.06 (0.91 to 1.22)	1.22 (1.13 to 1.31)*
Caribbean	35.93 (30.77–41.60)	388.69 (333.44–449.67)	43.49 (36.10–51.83)	361.03 (299.44–430.50)	0.21 (0.12 to 0.28)	−0.12 (−0.23 to 0.01)
Central Asia	39.61 (32.58–47.83)	240.57 (198.83–289.61)	84.08 (70.42–100.30)	342.57 (286.41–409.25)	1.12 (0.97 to 1.29)	1.18 (1.14 to 1.21)*
Central Europe	68.04 (55.34–82.36)	219.24 (177.92–265.8)	79.82 (64.12–97.87)	295.61 (234.84–365.66)	0.17 (0.12 to 0.23)	1.00 (0.96 to 1.05)*
Central Latin America	85.16 (65.16–110.12)	205.28 (157.96–264)	129.72 (101.79–163.75)	189.93 (148.94–239.86)	0.52 (0.39 to 0.66)	−0.28 (−0.36 to −0.20)*
Central Sub-Saharan Africa	16.71 (13.18–20.94)	141.23 (112.67–175.99)	45.14 (35.5–56.78)	144.15 (114.37–180.52)	1.70 (1.49 to 1.85)	0.08 (0.06 to 0.10)*
East Asia	290.33 (231.54–361.35)	87.74 (70.33–108.87)	345.23 (268.33–444.31)	100.45 (77.33–130.28)	0.19 (0.07 to 0.33)	0.55 (0.43 to 0.68)*
Eastern Europe	107.14 (85.53–135.12)	191.40 (152.34–241.66)	162.69 (127.44–206.21)	319.69 (247.7–407.74)	0.52 (0.47 to 0.58)	1.78 (1.65 to 1.91)*
Eastern Sub-Saharan Africa	85.03 (71.81–100.23)	201.90 (171.22–237.63)	195.54 (161.39–234.82)	187.25 (155.14–224.46)	1.30 (1.20 to 1.39)	−0.18 (−0.24 to −0.13)*
High-income Asia Pacific	111.09 (89.38–139.40)	243.11 (195.54–305.07)	110.61 (87.84–140.47)	285.56 (225.06–365.12)	0.01 (−0.07 to 0.06)	0.51 (0.41 to 0.61)*
High-income North America	402.65 (337.77–479.50)	532.60 (445.68–635.02)	559.16 (472.38–666.40)	648.97 (547.5–773.97)	0.39 (0.31 to 0.46)	0.59 (0.50 to 0.67)*
North Africa and Middle East	197.17 (157.25–242.31)	263.53 (212.30–321.81)	481.42 (380.85–591.43)	301.05 (237.91–369.99)	1.44 (1.34 to 1.61)	0.39 (0.34 to 0.43)*
Oceania	3.13 (2.66–3.66)	203.87 (173.76–238.06)	6.52 (5.33–7.81)	188.62 (154.52–225.75)	1.08 (0.95 to 1.22)	−0.22 (−0.25 to −0.20)*
South Asia	549.39 (419.08–719.62)	219.24 (168.25–285.73)	1192.2 (913.02–1560.66)	242.62 (186.16–317.08)	1.17 (1.11 to 1.25)	0.33 (0.30 to 0.35)*
Southeast Asia	354.37 (271.15–453.14)	303.38 (234.27–385.86)	545.51 (418.32–707.95)	294.59 (225.3–382.97)	0.54 (0.47 to 0.62)	0.01 (−0.09 to 0.09)
Southern Latin America	33.83 (29.06–39.35)	273.78 (235.24–318.26)	52.89 (42.16–64.28)	300.71 (238.84–366.34)	0.56 (0.38 to 0.76)	0.37 (0.31 to 0.43)*
Southern Sub-Saharan Africa	27.05 (20.31–35.92)	214.54 (162.93–281.85)	46.08 (35.09–60.93)	213.15 (162.43–281.52)	0.70 (0.61 to 0.80)	0.01 (−0.02 to 0.05)
Tropical Latin America	125.84 (96.65–162.38)	322.49 (249.06–414.01)	237.62 (183.66–306.39)	384.64 (295.96–497.81)	0.89 (0.81 to 0.98)	0.72 (0.59 to 0.85)*
Western Europe	360.56 (310.29–419.21)	372.63 (320.20–433.80)	587.76 (486.32–710.10)	604.10 (496.95–732.81)	0.63 (0.56 to 0.72)	1.56 (1.49 to 1.63)*
Western Sub-Saharan Africa	71.63 (54.88–92.61)	175.19 (136.32–224.21)	201.36 (154.53–261.49)	177.83 (138.21–228.84)	1.81 (1.74 to 1.87)	0.01 (−0.03 to 0.05)

95% CI = 95% confidence interval, 95% UI = 95% uncertainty interval, SDI = sociodemographic index, T1DM = type 1 diabetes mellitus, WCBA = women of childbearing age.

* Indicates *P* < .05.

Globally, DALYs for T1DM increased by 33%, while ASDR decreased from 53.16 to 47.11 per 100,000 population, with an AAPC of −0.48% (Table S1, Supplemental Digital Content, https://links.lww.com/MD/Q103). The number of mortality due to T1DM increased by 19%, while the ASMR decreased from 0.70 to 0.54 per 100,000 population, with an AAPC of −0.92% (Table S1, Supplemental Digital Content, https://links.lww.com/MD/Q103). Notably, the ASDR was higher and ASMR lower in the WCBA group compared to the overall T1DM population (Figure S1B and C, Supplemental Digital Content, https://links.lww.com/MD/Q102). The global decrease in the AAPC for ASDR was more pronounced, while the decrease in AAPC for ASMR was less marked compared to the overall T1DM population (Figure S2B and C, Supplemental Digital Content, https://links.lww.com/MD/Q102).

### 3.2. Global trends by age subgroup

**Figure 1.**
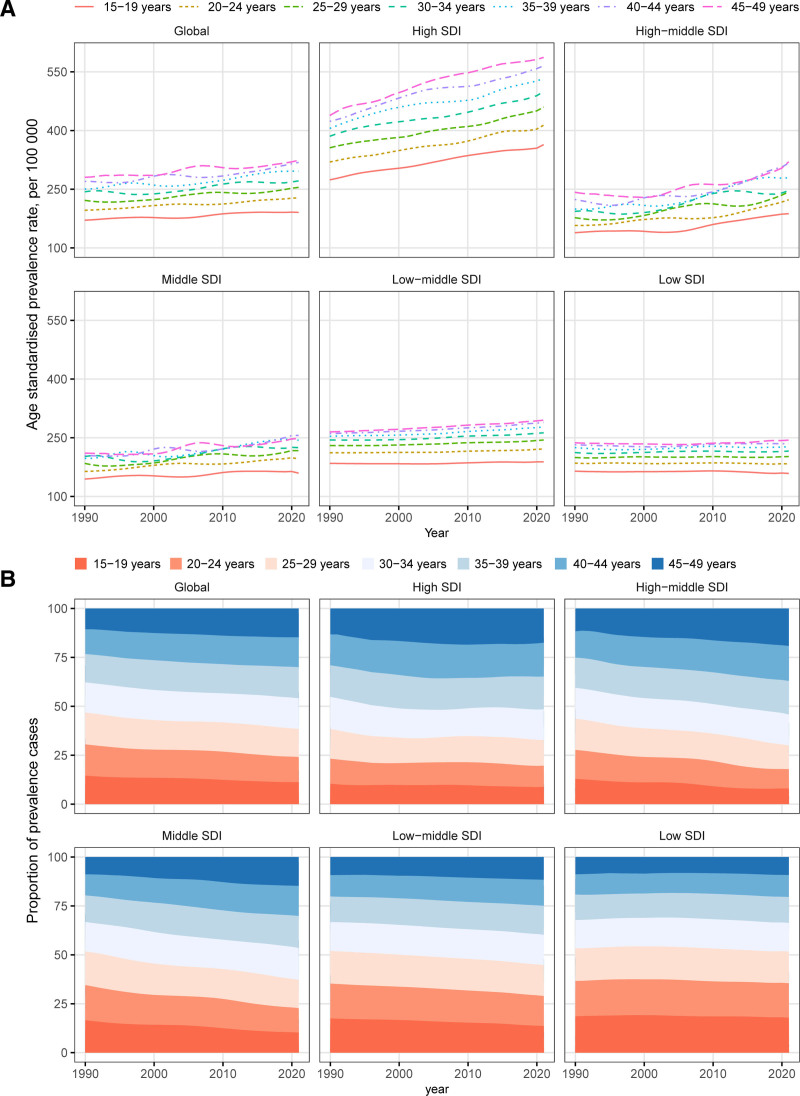
Temporal trend of age-standardized prevalence rate (A) and proportion of prevalent cases (B) of type 1 diabetes mellitus in women of childbearing age, by age groups, globally and by sociodemographic index, from 1990 to 2021.

From 1990 to 2021, the number of prevalent cases of T1DM in WCBA increased globally across all age groups (Figure S3A, Supplemental Digital Content, https://links.lww.com/MD/Q102). The ASPR also increased, with higher rates observed in older age groups (Fig. 1A). The proportion of prevalent cases increased most significantly in the 45 to 49 age group, while the 15 to 19 age group saw a decrease (Fig. 1B).

Similarly, global DALYs increased across all age groups during the same period (Figure S3B, Supplemental Digital Content, https://links.lww.com/MD/Q102). However, the global ASDR for T1DM in WCBA decreased, with the highest rates in the 40 to 44 age group and the lowest in the 15 to 19 age group (Figure S5A, Supplemental Digital Content, https://links.lww.com/MD/Q102). Older age groups showed a significant increase in the proportion of global DALYs, while younger age groups experienced a decrease (Figure S5B, Supplemental Digital Content, https://links.lww.com/MD/Q102). Similar trends were observed in ASMR across all age groups (Figure S3C, Supplemental Digital Content, https://links.lww.com/MD/Q102; Figure S6A and B, Supplemental Digital Content, https://links.lww.com/MD/Q102).

### 3.3. Global trends by SDI

From 1990 to 2021, the prevalent cases of T1DM in WCBA increased across all SDI subgroups (Figure S4A, Supplemental Digital Content, https://links.lww.com/MD/Q102). The percentage increase was higher as SDI levels decreased, with the largest increase of 146% in low SDI regions (Table 1). The ASPR also increased across all SDI subgroups, particularly in older age groups (Fig. 1A). In 2021, the ASPR in high SDI regions (479.89 per 100,000 population) was 2.32 times that of low SDI regions (206.21 per 100,000 population) (Table 1). Although ASPR was higher than the overall T1DM population across all SDI subgroups, the AAPC was lower (Figure S1A, Supplemental Digital Content, https://links.lww.com/MD/Q102; Figure S2A, Supplemental Digital Content, https://links.lww.com/MD/Q102). The proportion of prevalent cases increased in older age groups but decreased in younger ones across all SDI subgroups (Fig. 1B).

Regarding the number of DALYs and mortality, middle and lower SDI regions saw increases, while high and high-middle SDI regions experienced decreases (Figure S4B and C, Supplemental Digital Content, https://links.lww.com/MD/Q102). ASDR and ASMR for T1DM in WCBA decreased across all SDI subgroups, with the largest decrease in high-middle SDI region (AAPC of −1.23% for ASDR and −2.43% for ASMR) (Tables S1 and S2, Supplemental Digital Content, https://links.lww.com/MD/Q103). As SDI decreased, the proportion of DALYs in the 45 to 49 age group generally increased, particularly in high SDI region (Figure S5B, Supplemental Digital Content, https://links.lww.com/MD/Q102). Conversely, the proportion of mortality in the 15 to 19 age group showed a decreasing trend in high-middle, middle, and low-middle SDI regions (Figure S6B, Supplemental Digital Content, https://links.lww.com/MD/Q102).

### 3.4. Regional trends

**Figure 2.**
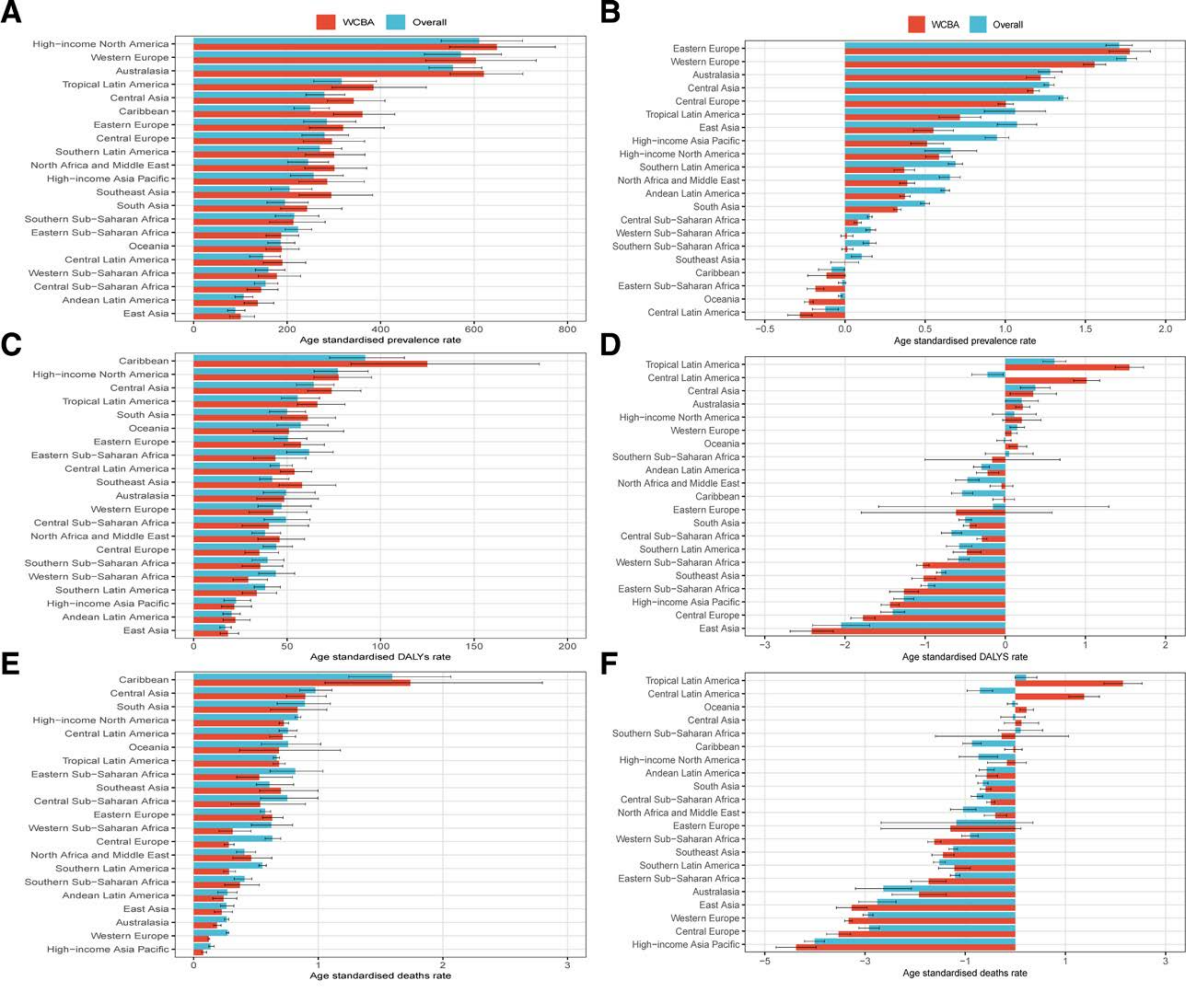
Age-standardized prevalence rate in 2021 (A), average annual percentage change in the age-standardized prevalence rate from 1990 to 2021 (B), age-standardized DALYs rate in 2021 (C), average annual percentage change in the age-standardized DALYs rate from 1990 to 2021 (D), age-standardized mortality rate in 2021 (E), and average annual percentage change in the age-standardized mortality rate from 1990 to 2021 (F) of type 1 diabetes mellitus in women of childbearing age for 21 regions, by sex. DALYs = disability-adjusted life years.

In 2021, high-income North America had the highest ASPR of T1DM in WCBA (648.97 per 100,000 population) but the lowest ASMR (0.08 per 100,000 population) among the 21 GBD regions (Table 1). The Caribbean had the highest ASDR (125.03 per 100,000 population) and ASMR (1.74 per 100,000 population) (Tables S1 and S2, Supplemental Digital Content, https://links.lww.com/MD/Q103). East Asia had the lowest ASPR (100.45 per 100,000 population) and ASDR (18.28 per 100,000 population) (Table 1; Table S1, Supplemental Digital Content, https://links.lww.com/MD/Q103). In most regions (18 out of 21), the ASPR of T1DM in WCBA was higher than in the overall T1DM population (Fig. 2A). Additionally, 11 regions had a higher ASDR for WCBA compared to the overall population (Fig. 2C), while 16 regions had a lower ASMR for WCBA (Fig. 2E). Eastern Europe had the largest average annual increase in ASPR (1.78%), while Tropical Latin America had the highest increases in ASDR (1.55%) and ASMR (2.15%) (Fig. 2B, D and F).

**Figure 3.**
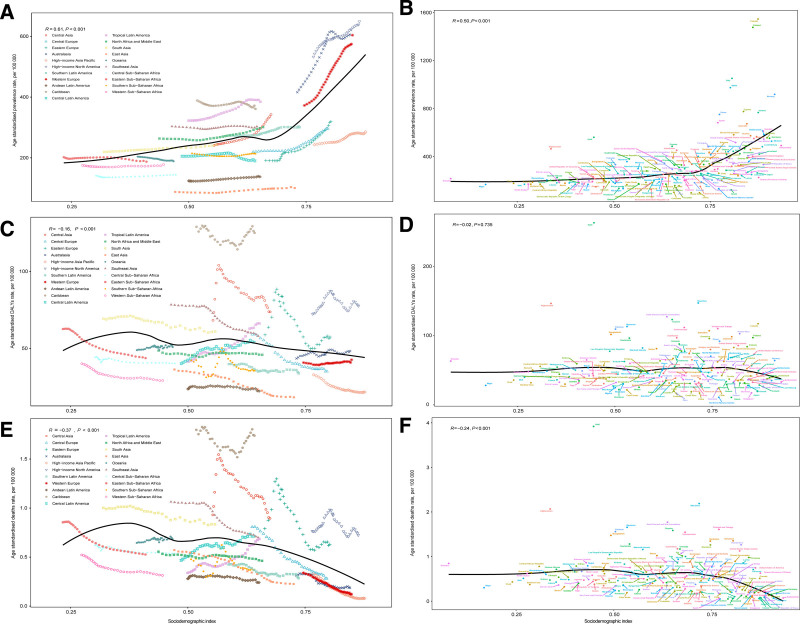
Age-standardized prevalence rate for 21 regions from 1990 to 2021 (A), age-standardized prevalence rate for 204 countries and territories in 2021 (B), age-standardized DALYs rate for 21 regions from 1990 to 2021 (C), age-standardized DALYs rate for 204 countries and territories in 2021 (D), age-standardized mortality rate for 21 regions from 1990 to 2021 (E), and age-standardized mortality rate for 204 countries and territories in 2021 (F) of type 1 diabetes mellitus in women of childbearing age, by sociodemographic index (SDI). Expected values based on the SDI and disease rate in all locations are shown as the black line. Each point shows the observed age-standardized rate for each location. DALYs = disability-adjusted life years.

Regionally, ASPR was significantly positively correlated with SDI from 1990 to 2021 (*P* < .05), with ASPR rising alongside SDI (Fig. 3A). High-income North America had a higher than expected ASPR based on SDI, while East Asia had a lower than expected ASPR (Fig. 3A). Conversely, SDI was negatively correlated with both ASDR and ASMR (*P* < .05). The Caribbean had a much higher than expected ASDR and ASMR, while Andean Latin America had much lower rates (Fig. 3C and E).

### 3.5. National trends

**Figure 4.**
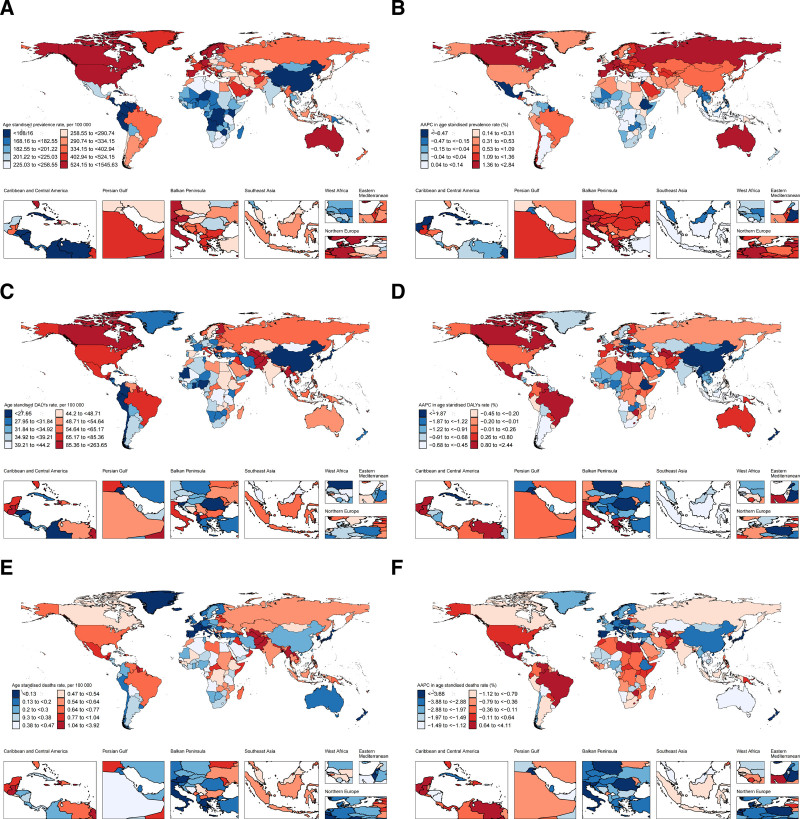
Age-standardized prevalence rate in 2021 (A), average annual percentage change in age-standardized prevalence rate from 1990 to 2021 (B), age-standardized DALYs rate in 2021 (C), average annual percentage change in age-standardized DALYs rate from 1990 to 2021 (D), age-standardized mortality rate in 2021 (E), and average annual percentage change in age-standardized mortality rate from 1990 to 2021 (F) of type 1 diabetes mellitus in women of childbearing age, by 204 countries and territories. DALYs = disability-adjusted life years.

At the national level in 2021, Canada had the highest ASPR of T1DM in WCBA at 1545.63 per 100,000 population, while Costa Rica had the lowest at 97.67 per 100,000 population (Table S3, Supplemental Digital Content, https://links.lww.com/MD/Q103; Fig. 4A). From 1990 to 2021, Czechia had the largest average annual increase in ASPR at 2.84%, and Saint Kitts and Nevis had the largest decrease at −1.13% (Table S3, Supplemental Digital Content, https://links.lww.com/MD/Q103; Fig. 4B). Haiti had the highest ASDR at 263.65 per 100,000 population, while Guam had the lowest at 10.72 per 100,000 population (Table S3, Supplemental Digital Content, https://links.lww.com/MD/Q103; Fig. 4C). Zimbabwe had the largest average annual increase in ASDR at 2.44%, and Saint Kitts and Nevis had the largest decrease at −3.87% (Table S3, Supplemental Digital Content, https://links.lww.com/MD/Q103; Fig. 4D). Haiti also had the highest ASMR at 3.92 per 100,000 population, with Singapore had the lowest at 0.02 per 100,000 population (Table S3, Supplemental Digital Content, https://links.lww.com/MD/Q103; Fig. 4E). Zimbabwe had the largest average annual increase in ASMR at 4.11% for, while Singapore had the largest average annual decrease at −7.45% (Table S3, Supplemental Digital Content, https://links.lww.com/MD/Q103; Fig. 4F).

Nationally, in 2021, ASPR was positively correlated with the SDI (*P* < .05), with Canada showing a higher burden than expected (Fig. 3B). Conversely, SDI was negatively correlated with ASDR and ASMR, with Haiti showing much higher than expected rates (Fig. 3D and F).

### 3.6. Risk factors

A detailed analysis of global data from 1990 to 2021 revealed high FPG, low temperature, and high temperature as the primary risk factors associated with DALYs for T1DM in WCBA. In 2021, these factors accounted for 47.11, 1.19, and 1.02 DALYs per 100,000 population, respectively. From 1990 to 2021, the corresponding AAPCs for these factors were −0.37%, −1.39%, and 0.99%. Notably, countries with a high-middle SDI levels, had the greatest reduction in burden associated with high FPG (AAPC −0.99%) and low temperature (−2.65%). Conversely, countries with high SDI experienced the highest burden due to high temperatures (AAPC 1.84%) (Table 2).

**Table 2:** Main risk factors for type 1 diabetes mellitus (T1DM) age-standardized related DALYs and average annual percentage change (AAPC) in women of childbearing age (WCBA) at global and sociodemographic index (SDI) levels, 1990–2021.

	1990		2021		1990–2021	
	Number of DALYs,000s (95% UI)	ASDR, per 100,000 (95% CI)	Number of DALYs,000s (95% UI)	ASDR, per 100,000 (95% CI)	Total percent change (95% UI)	AAPC in ASDR (%), from 1990 to 2021 (95% CI)
High fasting plasma glucose						
Global	692.30 (576.83 to 880.35)	53.15 (44.27 to 67.7)	923.59 (758.75 to 1112.9)	47.11 (38.72 to 56.72)	0.33 (0.07 to 0.47)	−0.37 (−0.46 to −0.28)*
High SDI	120.17 (102.49 to 142.62)	51.5 (43.81 to 61.27)	123.17 (98.05 to 156.17)	48.32 (38.45 to 61.33)	0.02 (−0.05 to 0.10)	−0.19 (−0.37 to −0.01)*
High-middle SDI	122.96 (104.50 to 146.49)	44.61 (37.82 to 53.28)	103.57 (83.12 to 130.40)	32.55 (26.23 to 40.86)	−0.16 (0.30 to −0.02)	−0.99 (−1.20 to −0.78)*
Middle SDI	203.95 (167.64 to 254.62)	47.86 (39.22 to 59.95)	264.88 (219.59 to 321.04)	42.23 (35.07 to 51.1)	0.30 (−0.01 to 0.46)	−0.39 (−0.45 to −0.33)*
Low-middle SDI	179.30 (138.28 to 264.15)	68.52 (52.81 to 101.72)	299.59 (236.95 to 367.93)	60.06 (47.48 to 73.83)	0.67 (0.17 to 0.97)	−0.42 (−0.49 to −0.35)*
Low SDI	64.98 (49.20 to 99.31)	61.66 (46.48 to 94.96)	131.34 (102.30 to 165.24)	49.96 (38.84 to 63.11)	1.02 (0.49 to 1.38)	−0.69 (−0.77 to −0.60)*
Low temperature						
Global	23.70 (16.93 to 31.69)	1.82 (1.30 to 2.43)	23.30 (14.61 to 34.61)	1.19 (0.75 to 1.77)	−0.02 (−0.25 to 0.20)	−1.39 (−2.04 to −0.74)*
High SDI	4.81 (4.09 to 5.52)	2.04 (1.74 to 2.34)	3.27 (2.75 to 3.87)	1.29 (1.09 to 1.53)	−0.32 (−0.37 to −0.27)	−1.46 (−1.90 to −1.01)*
High-middle SDI	5.64 (4.37 to 7.08)	2.03 (1.57 to 2.56)	2.78 (2.09 to 3.63)	0.90 (0.68 to 1.18)	−0.51 (−0.61 to −0.41)	−2.65 (−3.10 to −2.19)*
Middle SDI	7.18 (5.05 to 9.85)	1.68 (1.18 to 2.32)	6.43 (4.49 to 9.07)	1.03 (0.72 to 1.46)	−0.11 (−0.36 to 0.09)	−1.80 (−2.18 to −1.42)*
Low-middle SDI	4.37 (1.73 to 7.63)	1.66 (0.66 to 2.92)	7.86 (2.92 to 14.36)	1.57 (0.58 to 2.87)	0.80 (0.30 to 1.24)	−0.37 (−0.67 to −0.08)*
Low SDI	1.68 (0.95 to 2.83)	1.59 (0.89 to 2.70)	2.95 (1.72 to 4.50)	1.12 (0.65 to 1.71)	0.76 (0.17 to 1.35)	−1.53 (−1.77 to −1.28)*
High temperature						
Global	9.97 (3.00 to 18.18)	0.76 (0.23 to 1.39)	19.78 (5.47 to 37.94)	1.02 (0.28 to 1.95)	0.98 (0.36 to 1.39)	0.99 (0.67 to 1.30)*
High SDI	0.46 (−0.23 to 1.32)	0.20 (−0.1 to 0.57)	0.77 (−0.10 to 1.88)	0.31 (−0.04 to 0.75)	0.67 (−2.78 to 4.01)	1.84 (1.37 to 2.30)*
High-middle SDI	0.52 (−0.21 to 1.40)	0.19 (−0.07 to 0.51)	0.59 (−0.11 to 1.44)	0.19 (−0.04 to 0.47)	0.12 (−1.60 to 1.32)	0.18 (−0.64 to 1.01)
Middle SDI	2.82 (0.88 to 4.97)	0.66 (0.21 to 1.16)	4.67 (1.52 to 8.52)	0.75 (0.24 to 1.37)	0.66 (0.08 to 1.03)	0.45 (0.09 to 0.81)*
Low-middle SDI	5.02 (2.05 to 8.91)	1.92 (0.78 to 3.42)	11.00 (3.26 to 21.38)	2.20 (0.65 to 4.27)	1.19 (0.29 to 1.80)	0.52 (0.17 to 0.87)*
Low SDI	1.14 (0.47 to 2.08)	1.10 (0.45 to 2.01)	2.75 (0.93 to 5.06)	1.05 (0.35 to 1.92)	1.42 (0.64 to 2.19)	−0.22 (−0.54 to 0.11)

95% CI = 95% confidence interval, 95% UI = 95% uncertainty interval, ASDR = age-standardized DALYs rate, DALYs = disability-adjusted life years.

* Significant difference.

### 3.7. Predictive analysis

**Figure 5.**
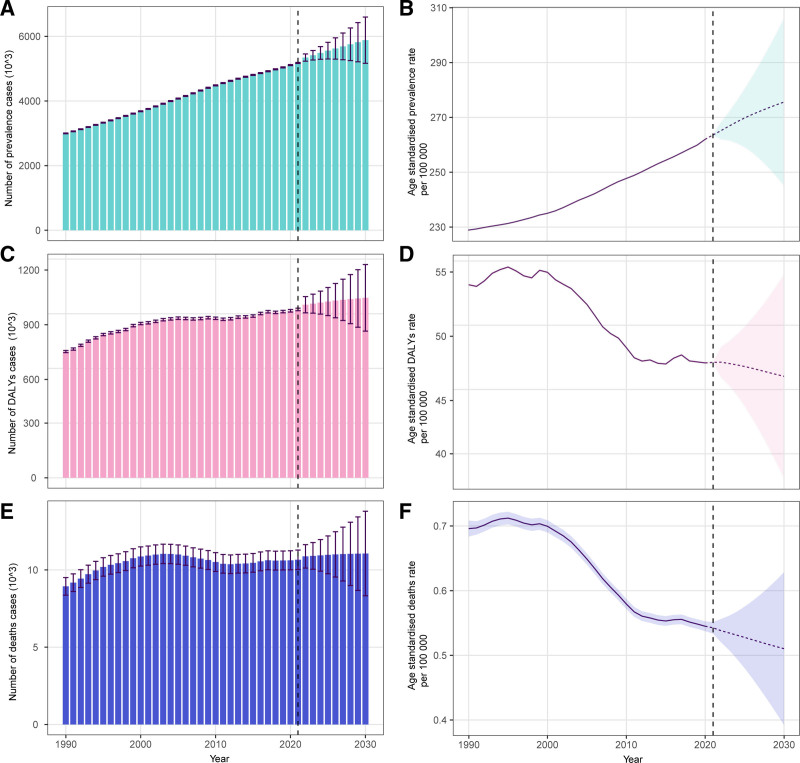
The predicted case number of prevalence to 2030 (A), the predicted age-standardized rate of prevalence to 2030 (B), the predicted case number of DALYs to 2030 (C), the predicted age-standardized rate of DALYs to 2030 (D), the predicted case number of mortality to 2030 (E), and the predicted age-standardized rate of mortality to 2030 (F) of type 1 diabetes mellitus in women of childbearing age globally. DALYs = disability-adjusted life years.

The predicted case number and ASR of prevalence, DALYs, and mortality for T1DM in WCBA to 2030 were illustrated in Figure 5. Globally, the case number of prevalence was predicted to increase (Fig. 5A), whereas the case number of DALYs and mortality was predicted to decrease annually to 2030 (Fig. 5B and C). Similarity, the prediction for the ASR of prevalence, DALYs, and mortality showed the same patterns.

## 4. Discussion

### 4.1. Principal findings

From 1990 to 2021, the global ASPR of T1DM in WCBA increased by 15%, indicating an increasing health burden. Notably, WCBA showed a higher ASDR but a lower ASMR compared to the overall T1DM population, reflecting the varying impacts of healthcare interventions. The focus on WCBA is due to the unique health challenges they face, including pregnancy-related complications and the need for specialized management during childbearing years. Disparities in T1DM burden were evident across SDI regions, with substantial increases observed in lower SDI regions, primarily among older age groups. Key global risk factors included high FPG and temperature extremes variations. Regional data highlighted stark contrasts, such as Canada’s high prevalence and Haiti’s elevated ASDR and ASMR, suggesting the need for region-specific health strategies. Projections suggest a continued increase in prevalence by 2030, with potential reductions in DALYs and mortality may due to medical advancements.

### 4.2. Comparison with other studies

From 1990 to 2021, the global prevalence cases of T1DM among WCBA increased significantly from 2.998 million to 5.18 million. This increase in disease burden is likely due to advancements in diagnostic technologies and lifestyle changes.^[13]^ Although the ASMR is lower than in the overall T1DM population, reflecting notable progress in diabetes management and treatment in reducing severe complications, the ASDR is higher than in the overall T1DM population. This suggests a greater disease burden for WCBA, possibly due to pregnancy-related complications and their unique physiological demands.^[14]^ The reduction in ASMR can be attributed to the widespread adoption of technologies such as continuous glucose monitoring and insulin therapy, which have effectively reduced life-threatening complications.^[15]^ Nonetheless, the higher ASDR indicates ongoing challenges in managing chronic and disabling aspects of the disease, particularly in the context of reproductive health. Temperature extremes have been identified as a significant risk factor for T1DM, particularly in WCBA, as fluctuations in temperature can trigger immune system responses, potentially worsening the autoimmune processes involved in T1DM development and progression.^[16]^ The autoimmune destruction of pancreatic beta cells is indeed a hallmark of T1DM, and this process is believed to be genetically predisposed. Specific genetic variations, particularly in immune-related genes such as those in the human leukocyte antigen region, have been implicated in increased susceptibility to T1DM.^[16]^ Genome-wide association studies have identified several single nucleotide polymorphisms that contribute to the risk of developing T1DM.^[17]^ Moreover, studies utilizing Mendelian randomization have provided insights into the causal relationships between specific genetic variants and the development of T1DM, further solidifying the genetic basis of the disease.^[18]^ Additionally, polygenic risk scores have been developed to predict individual susceptibility to T1DM by combining the effects of multiple genetic variants.^[19]^ Furthermore, low- and middle-income countries face significant barriers in implementing these advanced treatment technologies due to resource constraints and lack of infrastructure, resulting in disparate care outcomes across these regions.^[20]^ Therefore, more targeted public health interventions are urgently needed globally to ensure equitable access to effective diabetes care across different regions and populations.

Based on the study results, the ASPR of T1DM among WCBA has increased across all age groups, with the largest increase observed in the 45 to 49 age group. This trend may be related to the global tendency to delay childbearing and higher early-onset diabetes incidence and diagnosis rates.^[21]^ Conversely, the prevalence in the 15 to 19 age group has decreased, likely due to successful early intervention measures and educational programs targeting this age group.^[22]^ Despite the overall increase in prevalence, the ASDR and ASMR have decreased, which may reflect advancements in diabetes management, treatment technologies, and improved healthcare accessibility.^[23]^ However, the mortality rate remains the highest in the 40 to 44 age group, indicating that this cohort may be at increased risk for complications, necessitating more precise health management strategies.^[24]^ These findings align with previous research and highlight the importance of developing tailored strategies for different age groups. For example, more frequent screening and personalized medical plans may be necessary for older WCBA, whereas continuing early intervention and educational programs could help maintain or further reduce prevalence in younger populations.

Our study found that the prevalence of T1DM among WCBA increases as the SDI level decreases, with the largest increase observed in low-SDI regions. Although ASDR and ASMR have decreased across all SDI regions, the ASPR is significantly higher in high-SDI regions compared to low-SDI regions. The increase in T1DM prevalence in low-SDI regions may be attributed to a lack of medical resources and insufficient public health interventions.^[25]^ These areas often face inadequate healthcare infrastructure and limited access to essential management tools like insulin.^[26]^ Additionally, socioeconomic factors such as poverty, malnutrition, and low education levels exacerbate the risk and management challenges of T1DM.^[25]^ The higher ASPR in high-SDI regions may be due to more advanced diagnostic capabilities and greater disease awareness, allowing for earlier detection and management of T1DM cases.^[21]^ Furthermore, lifestyle factors such as high obesity rates and lack of physical activity may contribute to the rising prevalence.^[27]^ While ASDR and ASMR have decreased across all SDI regions, reflecting progress in diabetes management and treatment, the persistently high ASPR in high-SDI regions indicates that the risk and incidence of T1DM are still increasing, necessitating ongoing and targeted public health interventions.

Study found that the ASPR is highest in high-income North America, while the ASMR is the lowest. This can be attributed to the region’s developed healthcare system, higher health awareness, and effective diabetes management measures.^[28]^ In contrast, the Caribbean region has the highest ASDR and ASMR, likely due to limited healthcare resources, insufficient diabetes-related education and management, and adverse economic and social factors.^[29]^ The East Asia region has the lowest ASPR and ASDR, associated with healthy dietary habits and lifestyles, as well as progress made in diabetes management in the region.^[30]^ Moreover, this study also found that ASPR is positively correlated with the SDI, whereas ASDR and ASMR are negatively correlated with SDI. This indicates that with the improvement of socioeconomic development, the prevalence of diabetes increases, but the mortality rate and disease burden decrease. This may be because socioeconomic development brings better medical resources, higher health awareness, and more effective disease management measures.^[20]^

The results of this study indicate that Canada has the highest ASPR, while Costa Rica has the lowest, potentially attributable to differences in healthcare infrastructure and health awareness between the 2 countries.^[30]^ Haiti exhibits the highest ASDR and ASMR, which can be attributed to a lack of medical resources and socioeconomic challenges.^[20]^ In contrast, Guam and Singapore have the lowest ASDR and ASMR, benefiting from advanced healthcare systems and effective disease management.^[31]^ Significant differences are observed in the management of T1DM among WCBA across different countries, likely due to variations in healthcare policies and resource allocation.^[32]^ Furthermore, ASPR is positively correlated with the SDI, indicating that higher socioeconomic development levels are associated with increased reported diabetes prevalence. Conversely, ASDR and ASMR are negatively correlated with SDI, suggesting that with increased socioeconomic development, the burden of disability and mortality from the disease decreases.^[33]^

Fasting hyperglycemia remains a significant health risk for WCBA, closely linked to increased morbidity and mortality, particularly from diabetes and cardiovascular diseases.^[34]^ Despite some reductions in disease burden from fasting hyperglycemia between 1990 and 2021, the health impacts of temperature extremes have become more evident. Low temperature exposure has been associated with higher risks of cardiovascular and respiratory conditions, varying by region due to differences in healthcare infrastructure and public health preparedness.^[35]^ Conversely, the disease burden from high temperatures has risen, largely due to global warming and more frequent heatwaves, posing challenges for public health systems.^[36]^ Regional differences in managing climatic and metabolic factors highlight the need for tailored public health interventions. Advanced healthcare systems may better implement cooling measures and metabolic health management, reducing related health burdens.^[37]^ In contrast, resource-limited regions may face higher disease burdens due to inadequate responses to fasting hyperglycemia and temperature extremes.^[38]^

The projected increase in the global prevalence of T1DM among WCBA by 2030 can be attributed to factors such as improved diagnostic capabilities, heightened awareness, population growth, and aging.^[21]^ However, the decline in DALYs and mortality rates associated with T1DM suggests improvements in disease management, such as advancements in continuous glucose monitoring, insulin therapies, and comprehensive diabetes care programs.^[39]^ While earlier studies indicated high morbidity and mortality due to complications like cardiovascular disease and renal failure, recent data show positive trends with reduced incidence of these complications, likely due to better screening and the use of renoprotective agents.^[40]^ Public health initiatives and policies aimed at improving diabetes education and access to care, such as diabetes self-management education and support programs, have also played a crucial role in empowering individuals to manage their condition better, thereby improving quality of life and reducing disease burden.^[41]^

### 4.3. Strengths and limitations of the current study

This study is the first to comprehensively analyze the burden of T1DM in WCBA using GBD 2021 data, providing valuable insights into global, regional, and national trends. However, several limitations exist. Firstly, the results are influenced by the methodological limitations of GBD 2021. Data gaps and varied case definitions may have introduced biases, despite efforts to mitigate them. We used CIs instead of uncertainty intervals post age-standardization, necessitating cautious interpretation and verification through real-world studies. Secondly, limited epidemiological data, especially from low- and middle-income countries, may reduce accuracy. Variations in healthcare quality and socioeconomic factors likely led to underestimations in these regions. Lastly, the GBD data does not capture subnational variations, limiting intra-country analysis. Despite these limitations, the study offers the most up-to-date estimates on T1DM burden among WCBA, providing a foundation for targeted global health policies.

## 5. Conclusion

T1DM in WCBA represents a significant global public health challenge, contributing to a considerable disease burden. Although there have been decreases in DALYs and mortality rates in recent years, prevalence rates continue to increase, particularly in high-SDI regions and older age groups. There are substantial differences across various regions and countries globally. Despite the overall increase in the burden of T1DM, improvements in disease management and public health measures have contributed to reductions in DALYs and mortality rates to some extent. Therefore, enhancing diabetes education, improving access to healthcare services, and implementing effective management strategies are crucial for reducing the global burden of T1DM among WCBA. These insights are essential for policymakers in devising control measures and allocating resources to address the growing healthcare demands posed by T1DM.

## Acknowledgments

We would like to thank the staff of the Institute for Health Metrics and Evaluation and its collaborators who prepared these publicly available data.

## Author contributions

**Conceptualization:** Zhenhao Liu, Ling Zhu.

**Formal analysis:** Kui Wang.

**Resources:** Guangzhong Zeng.

**Software:** Guangzhong Zeng, Wei Pan.

**Supervision:** Wei Pan, Ling Zhu.

**Writing – original draft:** Zhenhao Liu.

**Writing – review & editing:** Ling Zhu.

## Supplementary Material


