# Supplementary material for: Global burden of type 1 diabetes mellitus in women of childbearing age from 1990 to 2021 with projections to 2030
Source: Medicine (Baltimore). 2025 Sep 19;104(38):e44419. doi: 10.1097/MD.0000000000044419 (PMC12459499; doi:10.1097/MD.0000000000044419)

Fig S1: Trends of age standardised prevalence, DALYs, and deaths rates of type 1 diabetes mellitus in women of childbearing age and overall from 1990 to 2021 at global and sociodemographic index levels.

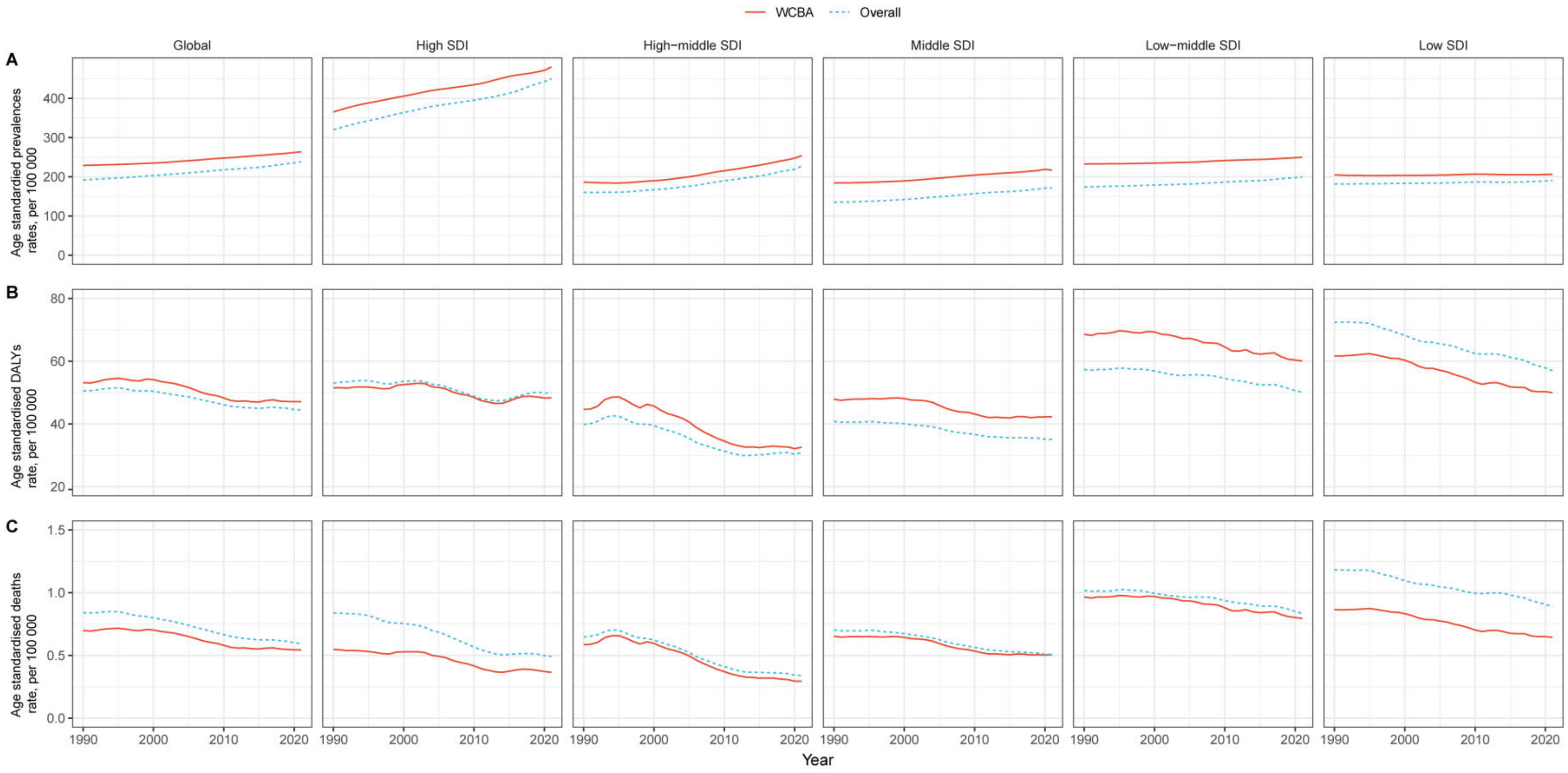

Fig S2: Average annual percent changes of age standardised prevalence, DALYs, and deaths rates of type 1 diabetes mellitus in women of childbearing age and overall from 1990 to 2021 at global and sociodemographic index levels.

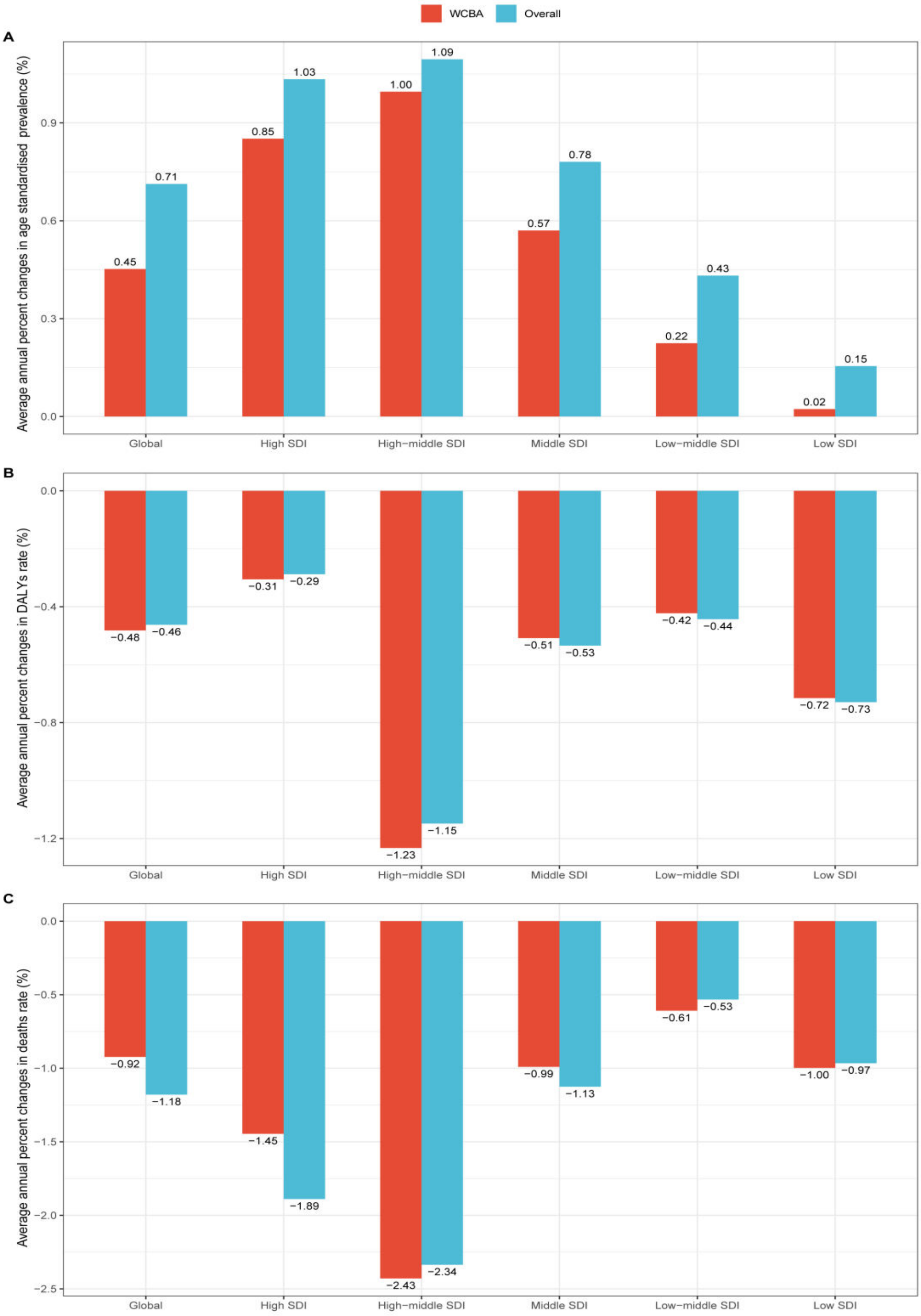

Fig S3: The number of percentage in prevalence, DALYs, and deaths of type 1 diabetes mellitus in women of childbearing age and overall at global levels from 1990 to 2021.

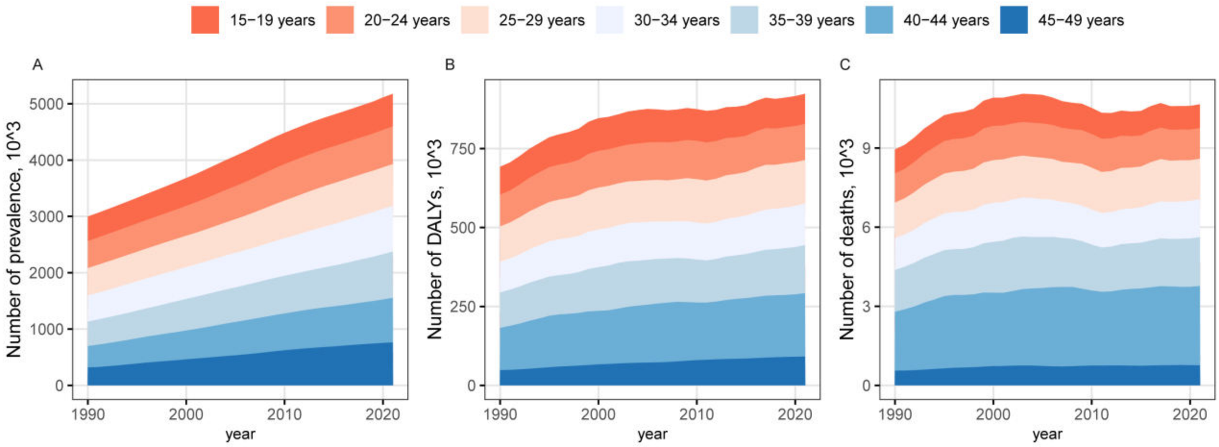

Fig S4: The number of percentage in prevalence, DALYs, and deaths of type 1 diabetes mellitus in women of childbearing age and overall at sociodemographic index levels from 1990 to 2021.

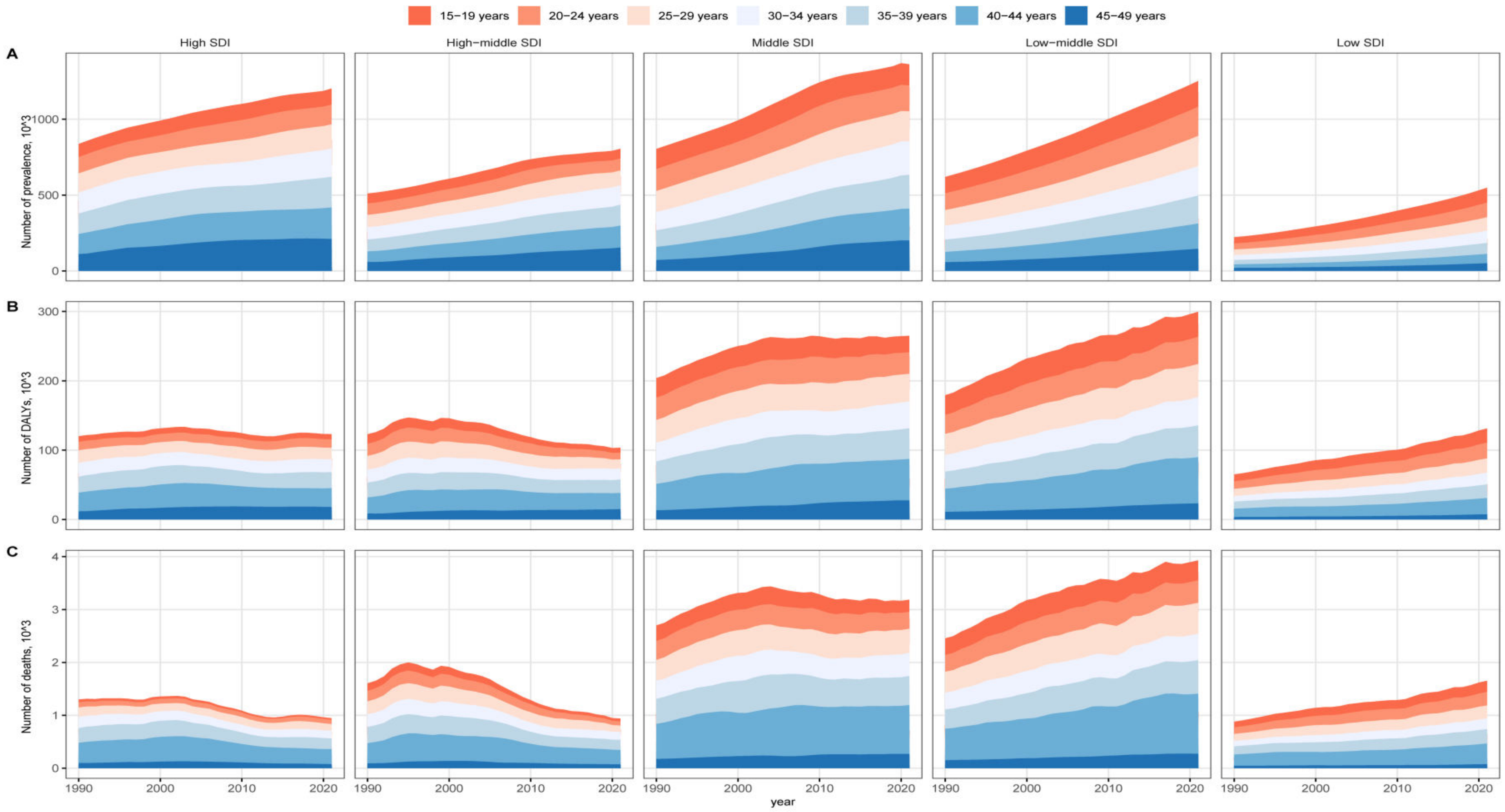

Fig S5: Temporal trend of age standardised DALYs (A) and proportion of DALYs cases (B) of type 1 diabetes mellitus in women of childbearing age, by age groups, globally and by sociodemographic index, from 1990 to 2021.

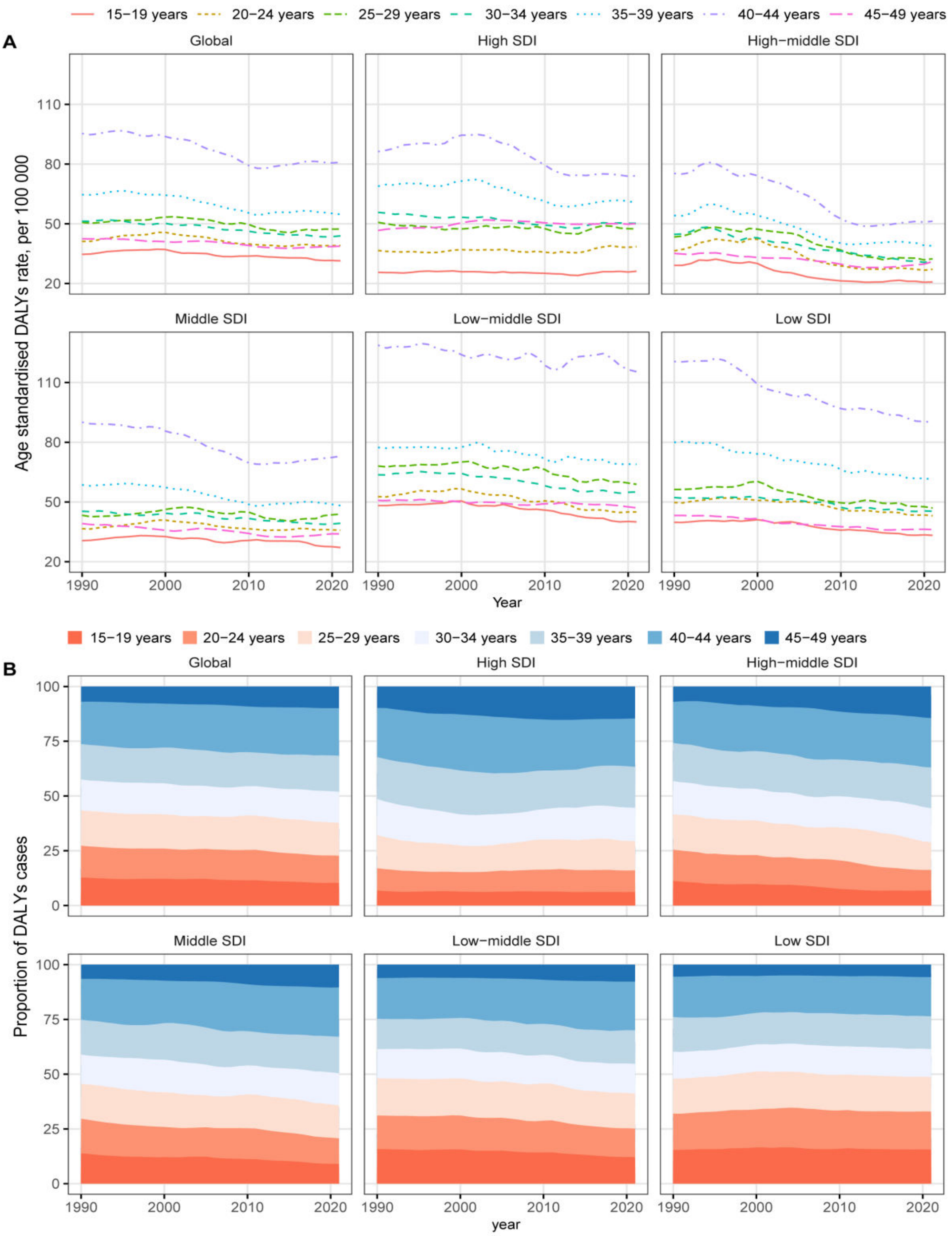

Fig S6: Temporal trend of age standardised deaths rate (A) and proportion of deaths cases (B) of type 1 diabetes mellitus in women of childbearing age, by age groups, globally and by sociodemographic index, from 1990 to 2021.

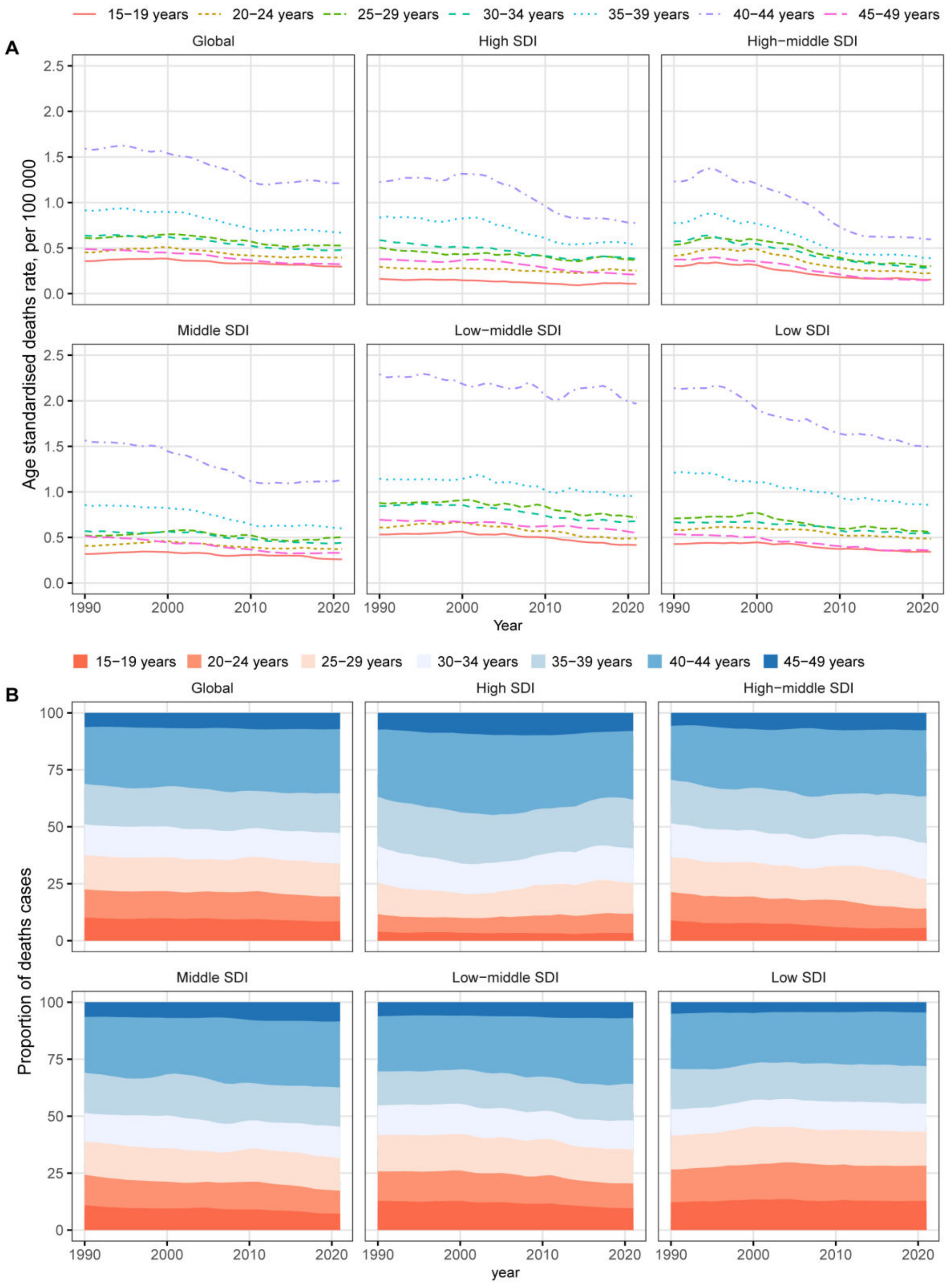

Supplement: Supplementary file 1 [file medi-104-e44419-s001.pdf]
