# Supplementary material for: Global burden of type 1 diabetes mellitus in women of childbearing age from 1990 to 2021 with projections to 2030
Source: Medicine (Baltimore). 2025 Sep 19;104(38):e44419. doi: 10.1097/MD.0000000000044419 (PMC12459499; doi:10.1097/MD.0000000000044419)
Supplement: Supplementary file 2 [file medi-104-e44419-s002.pdf]

Table S1: Age standardized DALYs rate (ASDR) of T1DM in WCBA in 1990 and 2021, and average annual percentage change (AAPC) from 1990 to 2021 at the global and regional level

	**1990**		**2021**		**1990-2021**	
	**DALYs, 000s (95% UI)**	**ASDRs per 100 000 (95% UI)**	**DALYs, 000s (95% UI)**	**ASDRs per 100 000 (95% UI)**	**Total percent change (95% UI)**	**AAPC, %, (95% CI)**
Global	692.47 (577.34 to 880.06)	53.16 (44.31 to 67.67)	923.57 (758.48 to 1112.64)	47.11 (38.7 to 56.71)	0.33 (0.07 to 0.47)	-0.48 (-0.66 to -0.31)*
SDI						
High	120.17 (102.51 to 142.58)	51.5 (43.82 to 61.25)	123.17 (98.06 to 156.19)	48.33 (38.45 to 61.34)	0.02 (-0.05 to 0.1)	-0.31 (-0.5 to -0.11)*
High-middle	122.99 (104.5 to 146.75)	44.62 (37.82 to 53.37)	103.59 (83.12 to 130.42)	32.56 (26.23 to 40.87)	-0.16 (-0.29 to -0.03)	-1.23 (-1.71 to -0.75)*
Middle	203.98 (167.76 to 254.84)	47.87 (39.25 to 60.02)	264.9 (219.68 to 321.15)	42.23 (35.08 to 51.12)	0.3 (-0.01 to 0.46)	-0.51 (-0.67 to -0.34)*
Low-middle	179.38 (138.3 to 264.43)	68.55 (52.81 to 101.86)	299.55 (237.39 to 367.11)	60.05 (47.57 to 73.66)	0.67 (0.16 to 0.98)	-0.42 (-0.48 to -0.37)*
Low	65.01 (49.28 to 99.78)	61.68 (46.54 to 95.41)	131.32 (102.35 to 165.28)	49.95 (38.86 to 63.09)	1.02 (0.49 to 1.39)	-0.72 (-0.81 to -0.62)*
Regions						
Andean Latin America	2.13 (1.57 to 2.86)	23.68 (17.42 to 31.94)	3.91 (2.76 to 5.28)	22.33 (15.75 to 30.19)	0.84 (0.45 to 1.28)	-0.22 (-0.36 to -0.08)*
Australasia	2.37 (1.83 to 3.05)	43.49 (33.47 to 56.12)	3.67 (2.56 to 5.06)	48.41 (33.79 to 66.78)	0.55 (0.33 to 0.76)	0.22 (0.13 to 0.31)*
Caribbean	11.62 (9.21 to 16.12)	128.95 (102.22 to 178.42)	15.01 (10.11 to 22.18)	125.03 (84.15 to 184.81)	0.29 (0 to 0.61)	-0.02 (-0.16 to 0.11)
Central Asia	11.14 (9.88 to 12.64)	68.28 (60.29 to 77.72)	18.03 (14.86 to 21.88)	73.78 (60.89 to 89.46)	0.62 (0.41 to 0.83)	0.35 (0.06 to 0.64)*
Central Europe	18.67 (16.78 to 21.19)	59.11 (53.02 to 67.15)	9.89 (7.72 to 12.78)	35.13 (27.44 to 45.43)	-0.47 (-0.54 to -0.39)	-1.78 (-1.93 to -1.63)*
Central Latin America	16.03 (13.73 to 19.06)	40.17 (34.42 to 47.76)	36.95 (31.7 to 43.29)	54 (46.34 to 63.24)	1.31 (1 to 1.59)	1.02 (0.85 to 1.18)
Central Sub-Saharan Africa	5.11 (3.26 to 7.98)	44.27 (28.32 to 69.6)	12.65 (8.13 to 19.25)	40.26 (25.95 to 61.45)	1.48 (0.82 to 2.28)	-0.29 (-0.35 to -0.23)*
East Asia	116.74 (89.95 to 150.01)	36.35 (27.85 to 47.05)	62.31 (48.05 to 82.25)	18.28 (14.14 to 24.05)	-0.47 (-0.62 to -0.28)	-2.42 (-2.69 to -2.15)*
Eastern Europe	32.78 (29.74 to 36.79)	58.86 (53.42 to 66.04)	28.31 (23.67 to 34.78)	57.41 (48.38 to 69.93)	-0.14 (-0.23 to -0.03)	-0.61 (-1.8 to 0.58)
Eastern Sub-Saharan Africa	25.36 (18.27 to 40.24)	62.64 (45.03 to 100.13)	45.25 (33.24 to 61.83)	43.75 (32.1 to 60.02)	0.78 (0.06 to 1.29)	-1.26 (-1.44 to -1.08)*
High-income Asia Pacific	15.22 (12.06 to 19.21)	32.95 (26.08 to 41.64)	8.73 (5.96 to 12.43)	21.72 (14.83 to 30.97)	-0.43 (-0.54 to -0.32)	-1.44 (-1.55 to -1.32)*
High-income North America	56.12 (47.97 to 67.03)	72.36 (61.57 to 86.78)	67.34 (55.85 to 82.67)	77.55 (64.33 to 95.19)	0.2 (0.15 to 0.26)	0.21 (-0.03 to 0.45)
North Africa and Middle East	36.32 (27.75 to 49.87)	48.61 (36.89 to 67.29)	73.71 (55.21 to 95.31)	45.89 (34.38 to 59.35)	1.03 (0.61 to 1.43)	-0.05 (-0.19 to 0.09)
Oceania	0.74 (0.45 to 1.22)	48.91 (30.24 to 80.81)	1.77 (1.1 to 2.79)	50.97 (31.73 to 80.25)	1.41 (0.66 to 2.32)	0.16 (0.05 to 0.27)*
South Asia	171.34 (130.16 to 245)	69.35 (52.55 to 99.71)	298.28 (229.2 to 370.85)	61.01 (46.82 to 75.92)	0.74 (0.24 to 1.06)	-0.44 (-0.52 to -0.37)*
Southeast Asia	89.1 (66.48 to 133.15)	78.42 (58.48 to 117.7)	108.01 (84.9 to 141.81)	58.04 (45.66 to 76.05)	0.21 (-0.22 to 0.51)	-1.02 (-1.17 to -0.87)*
Southern Latin America	4.9 (3.99 to 6.05)	39.85 (32.47 to 49.23)	6 (4.61 to 7.87)	33.83 (26 to 44.38)	0.23 (0.11 to 0.37)	-0.48 (-0.65 to -0.3)*
Southern Sub-Saharan Africa	5.33 (3.94 to 7.02)	42.28 (30.99 to 55.77)	7.71 (5.61 to 10.29)	35.64 (25.9 to 47.63)	0.45 (0.24 to 0.66)	-0.16 (-1 to 0.68)
Tropical Latin America	15.87 (12.45 to 20.6)	41.4 (32.46 to 53.79)	41.05 (34.32 to 50.25)	66.24 (55.53 to 80.96)	1.59 (1.41 to 1.79)	1.55 (1.37 to 1.72)*
Western Europe	39.83 (32.32 to 49.62)	40.8 (33.06 to 50.91)	42.39 (29.54 to 60.1)	42.62 (29.68 to 60.53)	0.06 (-0.08 to 0.21)	0.08 (0 to 0.15)*
Western Sub-Saharan Africa	15.74 (11.42 to 21.01)	40.15 (29.06 to 53.66)	32.6 (23.55 to 44.11)	29.2 (21.11 to 39.51)	1.07 (0.66 to 1.58)	-1.03 (-1.1 to -0.95)*

ASPR = age standardized DALYs rate; T1DM = T1DM=type 1 diabetes mellitus; WCBA = women of childbearing age; AAPC = average annual percentage change; SDI = socio-demographic index; 95% UI = 95% uncertainty interval; 95% CI = 95% confidence interval.

Table S2 Age standardized deaths rate (ASMR) of T1DM in WCBA in 1990 and 2021, and av erage annual percentage change (AAPC) from 1990 to 2021 at the global and regional level

	**1990**		**2021**		**1990-2021**	
	**Deaths, 000s (95% UI)**	**ASMRs per 100 000 (95% UI)**	**Deaths, 000s (95% UI)**	**ASMRs per 100 000 (95% UI)**	**Total percent change (95% UI)**	**AAPC, %, (95% CI)**
Global	8.95 (7.43 to 12.19)	0.70 (0.58 to 0.95)	10.66 (8.95 to 12.65)	0.54 (0.46 to 0.64)	0.19 (-0.10 to 0.37)	-0.92 (-1.18 to -0.67)*
SDI						
High	1.3 (1.25 to 1.35)	0.55 (0.53 to 0.57)	0.94 (0.89 to 1)	0.36 (0.34 to 0.39)	-0.27 (-0.3 to -0.24)	-1.45 (-1.81 to -1.08)*
High-middle	1.6 (1.37 to 1.92)	0.58 (0.5 to 0.7)	0.94 (0.82 to 1.1)	0.29 (0.26 to 0.34)	-0.42 (-0.54 to -0.3)	-2.43 (-2.99 to -1.87)*
Middle	2.7 (2.19 to 3.55)	0.65 (0.53 to 0.87)	3.19 (2.67 to 3.81)	0.5 (0.42 to 0.6)	0.18 (-0.17 to 0.37)	-0.99 (-1.23 to -0.75)*
Low-middle	2.46 (1.82 to 3.93)	0.97 (0.72 to 1.56)	3.93 (3.03 to 4.93)	0.79 (0.61 to 1)	0.6 (0.05 to 0.99)	-0.61 (-0.68 to -0.53)*
Low	0.88 (0.63 to 1.49)	0.86 (0.61 to 1.48)	1.65 (1.22 to 2.17)	0.64 (0.48 to 0.85)	0.88 (0.31 to 1.34)	-1 (-1.13 to -0.86)*
Regions						
Andean Latin America	0.02 (0.02 to 0.03)	0.28 (0.2 to 0.4)	0.04 (0.03 to 0.06)	0.24 (0.15 to 0.35)	0.72 (0.19 to 1.41)	-0.57 (-0.79 to -0.35)*
Australasia	0.02 (0.02 to 0.02)	0.31 (0.27 to 0.36)	0.01 (0.01 to 0.02)	0.19 (0.16 to 0.22)	-0.17 (-0.26 to -0.07)	-1.92 (-2.46 to -1.38)*
Caribbean	0.16 (0.12 to 0.23)	1.8 (1.38 to 2.64)	0.21 (0.13 to 0.34)	1.74 (1.05 to 2.8)	0.32 (-0.06 to 0.73)	-0.04 (-0.21 to 0.14)
Central Asia	0.15 (0.14 to 0.16)	0.93 (0.85 to 1.02)	0.22 (0.18 to 0.26)	0.89 (0.74 to 1.06)	0.48 (0.24 to 0.76)	0.12 (-0.22 to 0.47)
Central Europe	0.26 (0.24 to 0.27)	0.81 (0.76 to 0.86)	0.08 (0.07 to 0.09)	0.28 (0.24 to 0.32)	-0.69 (-0.72 to -0.64)	-3.53 (-3.77 to -3.29)*
Central Latin America	0.18 (0.17 to 0.19)	0.47 (0.44 to 0.5)	0.49 (0.42 to 0.56)	0.71 (0.61 to 0.82)	1.71 (1.32 to 2.1)	1.37 (1.07 to 1.68)*
Central Sub-Saharan Africa	0.07 (0.04 to 0.12)	0.62 (0.35 to 1.07)	0.16 (0.09 to 0.27)	0.53 (0.3 to 0.9)	1.38 (0.64 to 2.44)	-0.49 (-0.57 to -0.41)*
East Asia	1.79 (1.34 to 2.38)	0.57 (0.42 to 0.77)	0.78 (0.58 to 1.09)	0.23 (0.17 to 0.31)	-0.56 (-0.72 to -0.37)	-3.27 (-3.57 to -2.96)*
Eastern Europe	0.44 (0.42 to 0.47)	0.79 (0.75 to 0.84)	0.31 (0.27 to 0.35)	0.63 (0.55 to 0.72)	-0.31 (-0.4 to -0.21)	-1.29 (-2.68 to 0.11)*
Eastern Sub-Saharan Africa	0.33 (0.22 to 0.59)	0.85 (0.56 to 1.52)	0.53 (0.35 to 0.8)	0.53 (0.35 to 0.79)	0.61 (-0.18 to 1.26)	-1.73 (-2.08 to -1.38)*
High-income Asia Pacific	0.15 (0.12 to 0.19)	0.33 (0.27 to 0.4)	0.03 (0.03 to 0.04)	0.08 (0.06 to 0.1)	-0.79 (-0.83 to -0.73)	-4.38 (-4.78 to -3.98)*
High-income North America	0.61 (0.58 to 0.63)	0.76 (0.74 to 0.79)	0.63 (0.6 to 0.67)	0.72 (0.69 to 0.76)	0.05 (0 to 0.09)	-0.17 (-0.56 to 0.22)
North Africa and Middle East	0.41 (0.3 to 0.62)	0.55 (0.4 to 0.86)	0.75 (0.51 to 1.01)	0.46 (0.31 to 0.63)	0.84 (0.32 to 1.45)	-0.4 (-0.62 to -0.17)*
Oceania	0.01 (0.01 to 0.02)	0.64 (0.35 to 1.19)	0.02 (0.01 to 0.04)	0.68 (0.37 to 1.18)	1.5 (0.51 to 2.84)	0.23 (0.09 to 0.36)*
South Asia	2.41 (1.75 to 3.71)	1 (0.72 to 1.55)	4.05 (3 to 5.18)	0.83 (0.62 to 1.07)	0.68 (0.09 to 1.06)	-0.59 (-0.7 to -0.49)*
Southeast Asia	1.17 (0.83 to 1.97)	1.06 (0.75 to 1.81)	1.31 (0.99 to 1.88)	0.7 (0.53 to 1)	0.12 (-0.33 to 0.53)	-1.44 (-1.66 to -1.22)*
Southern Latin America	0.05 (0.04 to 0.06)	0.42 (0.37 to 0.49)	0.05 (0.04 to 0.06)	0.28 (0.24 to 0.34)	-0.02 (-0.11 to 0.1)	-1.22 (-1.53 to -0.9)*
Southern Sub-Saharan Africa	0.06 (0.04 to 0.08)	0.48 (0.33 to 0.66)	0.08 (0.05 to 0.11)	0.37 (0.25 to 0.53)	0.34 (0.06 to 0.69)	-0.27 (-1.59 to 1.06)
Tropical Latin America	0.13 (0.12 to 0.14)	0.34 (0.31 to 0.37)	0.43 (0.4 to 0.46)	0.69 (0.64 to 0.73)	2.32 (2.08 to 2.6)	2.15 (1.77 to 2.53)*
Western Europe	0.33 (0.32 to 0.35)	0.34 (0.32 to 0.36)	0.12 (0.12 to 0.13)	0.12 (0.12 to 0.13)	-0.63 (-0.65 to -0.62)	-3.33 (-3.41 to -3.26)*
Western Sub-Saharan Africa	0.2 (0.13 to 0.28)	0.52 (0.35 to 0.74)	0.34 (0.22 to 0.5)	0.31 (0.2 to 0.46)	0.74 (0.27 to 1.44)	-1.62 (-1.75 to -1.48)*

ASMR = age standardized deaths rate; T1DM = T1DM=type 1 diabetes mellitus; WCBA = women of childbearing age; AAPC = average annual percentage change; SDI = socio-demographic index; 95% UI = 95% uncertainty interval; 95% CI = 95% confidence interval.

Table S3 Age standardized prevalence, DALYs, and deaths rate of T1DM in WCBA in 1990 a nd 2021, and average annual percentage change (AAPC) from 1990 to 2021 by 204 countries and territories

	**Age standardized rate in 2021 (per 100 000 population) (95% UI)**			**AAPC in age standardized rate (%) from 1990 to 2021 (95% CI)**		
	**Prevalence**	**DALYs**	**Deaths**	**Prevalence**	**DALYs**	**Deaths**
Afghanistan	464.06 (363.21 to 581.54)	146.79 (81.81 to 260.63)	2.07 (0.93 to 4.13)	0.41 (0.31 to 0.51)	0.52 (0.4 to 0.65)	0.54 (0.41 to 0.68)
Albania	239.94 (184.89 to 305.84)	25.39 (17.22 to 36.45)	0.17 (0.11 to 0.26)	1.34 (1.28 to 1.4)	-0.48 (-0.76 to -0.21)	-2.5 (-3.61 to -1.38)
Algeria	250.69 (200.42 to 310.46)	38.67 (25.01 to 56.12)	0.41 (0.21 to 0.67)	0.32 (0.24 to 0.4)	-0.11 (-0.18 to -0.05)	-0.36 (-0.57 to -0.16)
American Samoa	150.92 (118.11 to 185.73)	27.7 (16.48 to 43.2)	0.32 (0.16 to 0.58)	0.26 (0.22 to 0.29)	1.17 (0.74 to 1.61)	1.67 (1.03 to 2.32)
Andorra	565.85 (439.1 to 712.79)	44.06 (28.98 to 63.94)	0.19 (0.11 to 0.31)	1.83 (1.75 to 1.91)	0.61 (0.51 to 0.72)	-1.81 (-2.38 to -1.23)
Angola	147.61 (115.67 to 184.26)	36.8 (21.65 to 59.38)	0.47 (0.23 to 0.84)	0.1 (0.07 to 0.13)	-0.45 (-0.6 to -0.29)	-0.78 (-1.16 to -0.41)
Antigua and Barbuda	384.07 (303.72 to 482.1)	80.15 (63.52 to 100.07)	0.93 (0.75 to 1.15)	-0.8 (-1.01 to -0.6)	-2.06 (-2.23 to -1.89)	-2.61 (-2.86 to -2.35)
Argentina	312.71 (247.15 to 383.15)	37.63 (28.67 to 49.6)	0.34 (0.28 to 0.41)	0.12 (0.01 to 0.22)	-0.52 (-0.72 to -0.32)	-1.07 (-1.43 to -0.71)
Armenia	334.1 (261.92 to 417.16)	65.17 (52.86 to 79.9)	0.76 (0.63 to 0.9)	0.73 (0.66 to 0.8)	-1.89 (-2.55 to -1.23)	-2.73 (-3.47 to -1.99)
Australia	672.87 (599.04 to 761.92)	52.4 (36.15 to 72.63)	0.2 (0.17 to 0.24)	1.37 (1.26 to 1.48)	0.52 (0.37 to 0.66)	-1.29 (-1.76 to -0.82)
Austria	572.74 (503.07 to 644.57)	41.79 (28.03 to 60.41)	0.09 (0.08 to 0.11)	2.04 (1.95 to 2.14)	0.23 (0.09 to 0.37)	-3.83 (-3.96 to -3.7)
Azerbaijan	357.68 (288.69 to 436.61)	74.14 (51.97 to 97.65)	0.89 (0.55 to 1.25)	1.07 (1.02 to 1.13)	-0.24 (-0.5 to 0.02)	-0.57 (-1.05 to -0.08)
Bahamas	317.98 (255.41 to 384.32)	98.63 (72.99 to 130.09)	1.35 (0.95 to 1.87)	-0.64 (-0.78 to -0.49)	-1.77 (-2.17 to -1.37)	-1.84 (-2.48 to -1.2)
Bahrain	329 (250.89 to 416.12)	49.96 (33.18 to 71.74)	0.49 (0.28 to 0.78)	0.31 (0.26 to 0.35)	-1 (-1.13 to -0.87)	-1.62 (-1.99 to -1.25)
Bangladesh	318.63 (260.06 to 379.3)	101.98 (62.72 to 156.59)	1.47 (0.78 to 2.45)	0.27 (0.23 to 0.31)	-0.57 (-0.88 to -0.27)	-0.73 (-1.16 to -0.3)
Barbados	435.17 (348.47 to 537.97)	107.3 (81.01 to 141.27)	1.35 (0.96 to 1.85)	-0.69 (-0.87 to -0.5)	-1.53 (-1.96 to -1.11)	-1.83 (-2.36 to -1.31)
Belarus	220.94 (170.7 to 281.09)	62.76 (48.61 to 80.84)	0.84 (0.63 to 1.09)	1.17 (1.04 to 1.3)	-0.27 (-1.13 to 0.6)	-0.51 (-1.67 to 0.66)
Belgium	456.54 (352.55 to 588.45)	35.9 (23.2 to 53.44)	0.12 (0.1 to 0.14)	1.32 (1.28 to 1.36)	-0.49 (-0.64 to -0.34)	-4.26 (-4.59 to -3.92)
Belize	344.73 (284.54 to 413.75)	81.36 (62.67 to 104.62)	1.02 (0.77 to 1.33)	-0.48 (-0.65 to -0.31)	-0.66 (-0.92 to -0.4)	-0.73 (-1.1 to -0.36)
Benin	177.42 (139.31 to 223.44)	30 (17.95 to 49.13)	0.32 (0.14 to 0.61)	-0.09 (-0.18 to 0)	-0.76 (-0.97 to -0.55)	-1.2 (-1.52 to -0.88)
Bermuda	239.35 (189.12 to 297.05)	35 (25.54 to 47.82)	0.31 (0.23 to 0.4)	-0.35 (-0.55 to -0.16)	-2.48 (-2.65 to -2.31)	-3.66 (-4.03 to -3.29)
Bhutan	207.71 (164.52 to 256.59)	49.31 (28.56 to 78.27)	0.67 (0.32 to 1.18)	0.25 (0.24 to 0.27)	-1.07 (-1.27 to -0.87)	-1.43 (-1.64 to -1.22)
Bolivia (Plurinational State of)	183.2 (138.4 to 229.29)	34.21 (19.49 to 56.62)	0.4 (0.17 to 0.78)	0.06 (-0.02 to 0.14)	-0.83 (-0.91 to -0.74)	-1.24 (-1.42 to -1.05)
Bosnia and Herzegovina	334.62 (265.67 to 409.88)	47.78 (33.03 to 66.61)	0.46 (0.27 to 0.71)	0.86 (0.79 to 0.92)	-1.39 (-1.84 to -0.95)	-1.96 (-2.87 to -1.04)
Botswana	238.51 (185.03 to 305.69)	31.59 (19.05 to 50.2)	0.26 (0.11 to 0.51)	0.08 (0.04 to 0.12)	-0.4 (-1.1 to 0.31)	-0.89 (-2.23 to 0.46)
Brazil	388.57 (298.32 to 503.85)	67.13 (56.17 to 82.12)	0.7 (0.65 to 0.75)	0.73 (0.6 to 0.86)	1.58 (1.4 to 1.76)	2.2 (1.81 to 2.58)
Brunei Darussalam	581.17 (494.93 to 671.71)	82.96 (56.43 to 118.71)	0.89 (0.51 to 1.42)	-0.2 (-0.23 to -0.16)	-2.16 (-2.3 to -2.01)	-2.97 (-3.18 to -2.76)
Bulgaria	443.2 (343.32 to 561.84)	62.2 (47.26 to 81.68)	0.6 (0.46 to 0.76)	0.89 (0.8 to 0.99)	-1.05 (-1.4 to -0.69)	-1.92 (-2.39 to -1.45)
Burkina Faso	169.23 (131.83 to 214.66)	31.96 (19.47 to 53.03)	0.37 (0.18 to 0.71)	-0.15 (-0.22 to -0.08)	-1.46 (-1.61 to -1.31)	-1.92 (-2.17 to -1.67)
Burundi	185.81 (154.8 to 222.88)	50.35 (28.97 to 81.94)	0.64 (0.28 to 1.19)	-0.29 (-0.33 to -0.24)	-0.77 (-0.93 to -0.61)	-1.02 (-1.31 to -0.73)
Cabo Verde	165.55 (131.16 to 205.63)	21.7 (12.59 to 33.66)	0.19 (0.08 to 0.37)	0.1 (0.05 to 0.15)	-1.01 (-1.18 to -0.84)	-1.86 (-2.14 to -1.59)
Cambodia	237.69 (187.6 to 295.96)	57.28 (35.69 to 91.03)	0.75 (0.38 to 1.32)	-0.06 (-0.24 to 0.11)	-1.2 (-1.4 to -1)	-1.57 (-1.84 to -1.29)
Cameroon	189.76 (150.9 to 236.75)	37.39 (20.96 to 63.23)	0.44 (0.19 to 0.89)	-0.03 (-0.06 to -0.01)	-1 (-1.03 to -0.97)	-1.52 (-1.65 to -1.4)
Canada	1545.63 (1412.89 to 1672.28)	117.44 (85.34 to 157.34)	0.51 (0.43 to 0.6)	1.7 (1.56 to 1.84)	0.81 (0.5 to 1.13)	-1.12 (-1.83 to -0.39)
Central African Republic	169.42 (130.98 to 215.76)	55.57 (31.78 to 94.41)	0.78 (0.37 to 1.48)	-0.04 (-0.08 to -0.01)	0.03 (-0.05 to 0.12)	0.03 (-0.17 to 0.23)
Chad	176.2 (137.11 to 223.98)	35.85 (20.67 to 62.11)	0.43 (0.2 to 0.88)	0 (-0.02 to 0.01)	-0.04 (-0.1 to 0.02)	-0.15 (-0.3 to 0.01)
Chile	260.11 (203.04 to 324.86)	24.45 (17.41 to 34.23)	0.16 (0.13 to 0.2)	1.09 (1.05 to 1.13)	-0.54 (-0.68 to -0.4)	-2.24 (-2.51 to -1.96)
China	97.75 (74.8 to 127.43)	17.1 (12.95 to 22.83)	0.21 (0.15 to 0.3)	0.58 (0.45 to 0.72)	-2.56 (-2.81 to -2.3)	-3.42 (-3.72 to -3.13)
Colombia	101.7 (80.33 to 128.69)	19.77 (14.71 to 26.55)	0.22 (0.16 to 0.28)	0.01 (-0.07 to 0.09)	0.01 (-0.27 to 0.29)	-0.12 (-0.58 to 0.34)
Comoros	197.09 (164.6 to 235.37)	49.84 (27.62 to 85.51)	0.62 (0.27 to 1.22)	-0.03 (-0.07 to 0)	-0.65 (-1.35 to 0.07)	-0.94 (-1.9 to 0.04)
Congo	184 (144.88 to 229.01)	49.47 (27.72 to 83.82)	0.65 (0.31 to 1.26)	0.02 (-0.04 to 0.09)	-0.02 (-0.32 to 0.28)	-0.15 (-0.52 to 0.22)

Cook Islands	201.26 (160.02 to 245.73)	42.3 (25.96 to 66.07)	0.54 (0.26 to 0.94)	-0.21 (-0.28 to -0.14)	-1.3 (-1.55 to -1.05)	-1.71 (-2.03 to -1.39)
Costa Rica	97.67 (76.65 to 124.37)	19.09 (14.4 to 25.14)	0.22 (0.17 to 0.27)	-0.07 (-0.2 to 0.07)	-0.07 (-0.44 to 0.29)	-0.11 (-0.75 to 0.53)
Croatia	185.81 (145.28 to 236.16)	32.18 (19.02 to 51.06)	0.35 (0.16 to 0.66)	0.03 (-0.02 to 0.08)	-0.19 (-0.53 to 0.15)	-0.4 (-0.93 to 0.12)
Cuba	385.13 (306.41 to 468.84)	37.22 (25.88 to 51.8)	0.21 (0.16 to 0.25)	1.8 (1.72 to 1.88)	-0.93 (-1.1 to -0.76)	-3.4 (-3.61 to -3.19)
Cyprus	156.16 (123.67 to 195.13)	28.57 (21.49 to 37.93)	0.31 (0.24 to 0.39)	-1.08 (-1.27 to -0.89)	-2.9 (-3.31 to -2.48)	-3.75 (-4.38 to -3.13)
Czechia	774.64 (676.47 to 890.75)	58.97 (40.19 to 82.16)	0.24 (0.15 to 0.38)	2.84 (2.75 to 2.93)	-0.55 (-1.19 to 0.1)	-3.95 (-4.47 to -3.43)
Côte d'Ivoire	329.76 (257.91 to 417.41)	36.28 (25.95 to 50.01)	0.26 (0.2 to 0.32)	1.12 (0.99 to 1.25)	-1.21 (-1.39 to -1.03)	-3.13 (-3.53 to -2.72)
Democratic People's Republic of Korea	121.27 (97.44 to 149.8)	46.92 (26.67 to 77.2)	0.72 (0.35 to 1.27)	0.31 (0.21 to 0.4)	-0.26 (-0.35 to -0.18)	-0.36 (-0.45 to -0.28)
Democratic Republic of the Congo	137.52 (108.95 to 173.07)	40.06 (22.47 to 67.12)	0.54 (0.25 to 1.01)	0.06 (0.03 to 0.1)	-0.17 (-0.35 to 0.02)	-0.33 (-0.56 to -0.1)
Denmark	555.9 (429.89 to 707.38)	37.67 (24.52 to 56.06)	0.13 (0.11 to 0.16)	1.09 (1.02 to 1.16)	-0.91 (-1.12 to -0.7)	-4.63 (-5.21 to -4.05)
Djibouti	186.28 (154.83 to 224.09)	39.65 (22.31 to 64.63)	0.46 (0.2 to 0.9)	-0.16 (-0.2 to -0.11)	-0.09 (-0.36 to 0.18)	-0.12 (-0.49 to 0.25)
Dominica	338.92 (279.45 to 406.02)	95.93 (63.03 to 140.38)	1.26 (0.72 to 2.02)	-0.57 (-0.75 to -0.39)	-0.33 (-0.47 to -0.19)	-0.36 (-0.55 to -0.18)
Dominican Republic	287.31 (231.23 to 351.09)	63.67 (40.67 to 94.08)	0.76 (0.4 to 1.26)	-0.16 (-0.22 to -0.11)	-0.04 (-0.37 to 0.29)	0 (-0.47 to 0.47)
Ecuador	139.96 (110.45 to 175.27)	26.53 (19.14 to 35.44)	0.32 (0.22 to 0.44)	0.27 (0.23 to 0.31)	0.36 (0.14 to 0.59)	0.41 (0.09 to 0.74)
Egypt	298.08 (229.33 to 380.29)	46.55 (30.35 to 68.2)	0.47 (0.25 to 0.78)	0.5 (0.43 to 0.56)	1.18 (0.45 to 1.91)	1.87 (0.75 to 3)
El Salvador	173.62 (142.59 to 206.9)	40.11 (24.7 to 60.01)	0.5 (0.25 to 0.82)	0.34 (0.32 to 0.37)	0.17 (-0.26 to 0.6)	-0.18 (-0.35 to 0)
Equatorial Guinea	157.31 (123.3 to 197.42)	33.65 (18.18 to 57.58)	0.4 (0.16 to 0.8)	0.47 (0.38 to 0.56)	-1.44 (-1.56 to -1.33)	-2.19 (-2.35 to -2.02)
Eritrea	210.25 (173.78 to 254.68)	59.49 (34.39 to 97.64)	0.79 (0.37 to 1.47)	0.04 (0.01 to 0.06)	0.02 (-0.28 to 0.32)	-0.1 (-0.46 to 0.26)
Estonia	388.25 (305.18 to 490.92)	84.2 (68.42 to 103.65)	0.98 (0.79 to 1.19)	1.01 (0.89 to 1.14)	0.35 (-0.41 to 1.11)	-0.17 (-1.33 to 0.99)
Eswatini	278.8 (208.31 to 370.2)	47.06 (25.38 to 79.26)	0.48 (0.17 to 0.99)	0.03 (0.01 to 0.05)	0.46 (0.07 to 0.85)	0.78 (0.16 to 1.4)
Ethiopia	183.63 (153.41 to 222.41)	44.26 (28.34 to 64.51)	0.54 (0.28 to 0.89)	-0.48 (-0.59 to -0.36)	-2.88 (-3.31 to -2.45)	-3.67 (-4.29 to -3.04)
Fiji	329.2 (271.17 to 397.1)	77.75 (46.76 to 122.7)	1.01 (0.51 to 1.77)	-0.64 (-0.87 to -0.42)	-0.47 (-0.61 to -0.33)	-0.45 (-0.65 to -0.25)
Finland	1476.28 (1361.54 to 1609.79)	97.26 (65.88 to 136.05)	0.19 (0.16 to 0.23)	1.19 (0.88 to 1.5)	0.38 (0.2 to 0.55)	-3.28 (-3.61 to -2.95)
France	465.48 (391.43 to 554.43)	31.62 (21.49 to 45.67)	0.11 (0.09 to 0.13)	2.04 (2 to 2.08)	0.52 (0.37 to 0.67)	-2.47 (-2.69 to -2.25)
Gabon	169.55 (132.72 to 210.76)	34.96 (19.71 to 58.46)	0.42 (0.18 to 0.81)	0.11 (0.07 to 0.15)	-0.69 (-0.85 to -0.53)	-1.06 (-1.27 to -0.85)
Gambia	180.56 (140.07 to 228.12)	34.6 (19.87 to 59.76)	0.4 (0.17 to 0.84)	0.06 (0.04 to 0.09)	-0.04 (-0.35 to 0.28)	-0.17 (-0.65 to 0.31)
Georgia	333.17 (261.75 to 410.86)	62.6 (50.6 to 77.08)	0.71 (0.6 to 0.84)	1.03 (0.96 to 1.1)	0.26 (-0.51 to 1.04)	-0.05 (-1.01 to 0.92)
Germany	552.03 (445.66 to 668.65)	39.16 (26.91 to 55.73)	0.15 (0.13 to 0.18)	1.86 (1.77 to 1.94)	-0.11 (-0.21 to 0)	-3.21 (-3.43 to -2.99)
Ghana	169.85 (136.29 to 210.15)	25.44 (15.1 to 39.67)	0.26 (0.12 to 0.48)	0.04 (0.02 to 0.07)	-0.87 (-0.94 to -0.81)	-1.47 (-1.57 to -1.37)
Greece	415.37 (321.19 to 533.92)	29.4 (18.01 to 45.37)	0.05 (0.04 to 0.06)	2.18 (2.07 to 2.28)	1.15 (1 to 1.29)	-3.09 (-3.2 to -2.97)
Greenland	423.32 (330.48 to 538.83)	31.49 (20.24 to 46.8)	0.13 (0.07 to 0.21)	0.32 (0.25 to 0.38)	-0.69 (-0.78 to -0.6)	-2.88 (-3.42 to -2.34)
Grenada	307.93 (247 to 370.89)	88.04 (66.95 to 113.63)	1.17 (0.86 to 1.55)	-0.84 (-0.93 to -0.75)	-2.2 (-2.52 to -1.87)	-2.61 (-3.04 to -2.17)
Guam	121.18 (95.23 to 154.13)	10.72 (6.51 to 16.27)	0.06 (0.03 to 0.1)	0.15 (0 to 0.3)	-0.43 (-0.86 to 0.01)	-1.34 (-2.46 to -0.2)
Guatemala	379.46 (318.3 to 444.46)	96.63 (76.06 to 120.55)	1.28 (0.97 to 1.63)	1.33 (1.07 to 1.59)	1.51 (1.23 to 1.78)	1.66 (1.28 to 2.04)
Guinea	201.6 (157.12 to 255.71)	40.05 (23.37 to 67.12)	0.47 (0.22 to 0.91)	-0.07 (-0.1 to -0.04)	-0.44 (-0.56 to -0.33)	-0.71 (-0.87 to -0.55)
Guinea-Bissau	224.17 (172.09 to 283.38)	54.54 (31.12 to 96.34)	0.71 (0.33 to 1.44)	-0.06 (-0.08 to -0.04)	-0.78 (-0.83 to -0.73)	-1.07 (-1.2 to -0.93)
Guyana	484.79 (399.23 to 586.73)	123.44 (87.08 to 172.31)	1.59 (0.99 to 2.39)	-0.54 (-0.64 to -0.43)	-0.68 (-0.94 to -0.42)	-0.72 (-1.07 to -0.38)
Haiti	560.02 (453.7 to 672.11)	263.65 (143.14 to 459.56)	3.92 (1.81 to 7.47)	-0.47 (-0.53 to -0.41)	-0.52 (-0.69 to -0.35)	-0.61 (-0.8 to -0.41)
Honduras	134.45 (105.94 to 167.2)	22.22 (12.43 to 36.03)	0.23 (0.09 to 0.45)	0.54 (0.52 to 0.57)	-0.7 (-1 to -0.41)	-1.3 (-1.66 to -0.93)
Hungary	284.1 (224.53 to 354.29)	33.72 (24.62 to 45.21)	0.27 (0.21 to 0.33)	1.33 (1.2 to 1.46)	-1.83 (-2.06 to -1.6)	-3.45 (-3.82 to -3.07)
Iceland	419.39 (320.72 to 545.32)	28.62 (17.75 to 44.03)	0.07 (0.06 to 0.09)	1.05 (0.97 to 1.13)	0.13 (0.04 to 0.21)	-2.59 (-3.56 to -1.61)
India	220.96 (166.17 to 294.51)	48.45 (36.62 to 61.12)	0.64 (0.46 to 0.85)	0.27 (0.23 to 0.3)	-0.8 (-0.91 to -0.69)	-1.03 (-1.18 to -0.88)
Indonesia	326.79 (241.33 to 447.09)	55.57 (41.16 to 78.27)	0.61 (0.41 to 0.99)	0.13 (0.1 to 0.16)	-0.6 (-0.67 to -0.52)	-0.95 (-1.04 to -0.85)
Iran (Islamic Republic of)	260.42 (195.81 to 345.19)	30.93 (20.68 to 41.92)	0.24 (0.13 to 0.32)	0.39 (0.33 to 0.46)	-0.01 (-0.38 to 0.36)	-0.52 (-0.97 to -0.07)
Iraq	263.33 (213.55 to 322.75)	65.2 (38.87 to 103.77)	0.86 (0.43 to 1.5)	0.21 (0.15 to 0.28)	-1.24 (-1.4 to -1.08)	-1.58 (-1.78 to -1.39)
Ireland	766.09 (681.2 to 862.07)	49.29 (32.48 to 70.6)	0.08 (0.07 to 0.1)	2.79 (2.73 to 2.86)	1.45 (1.35 to 1.55)	-3.51 (-3.93 to -3.08)

Israel	443.07 (420.47 to 464.86)	32.01 (21.8 to 44.88)	0.1 (0.08 to 0.13)	1.19 (1.12 to 1.27)	-0.55 (-0.65 to -0.44)	-4.08 (-4.37 to -3.78)
Italy	1051.05 (800.65 to 1374.96)	68.56 (43.26 to 104.95)	0.07 (0.06 to 0.07)	1.94 (1.77 to 2.12)	0.98 (0.78 to 1.18)	-4.01 (-4.32 to -3.71)
Jamaica	298.15 (239.4 to 361.21)	77.36 (55.9 to 104.7)	1 (0.66 to 1.43)	-0.76 (-0.96 to -0.56)	-0.28 (-0.82 to 0.26)	-0.08 (-0.88 to 0.73)
Japan	262.9 (202.89 to 343.24)	19.32 (12.96 to 27.86)	0.06 (0.06 to 0.06)	0.56 (0.48 to 0.63)	-1 (-1.15 to -0.84)	-4.6 (-5.03 to -4.16)
Jordan	293.92 (225.02 to 382.34)	30.08 (19.89 to 43.6)	0.2 (0.11 to 0.34)	-0.98 (-1.26 to -0.7)	-2.48 (-2.64 to -2.32)	-4.08 (-4.67 to -3.5)
Kazakhstan	258.63 (209.06 to 319.92)	47.85 (39.09 to 59.43)	0.54 (0.47 to 0.63)	0.8 (0.67 to 0.94)	-0.81 (-1.15 to -0.47)	-1.3 (-1.81 to -0.79)
Kenya	146.87 (118.82 to 182.62)	30.75 (21.13 to 45.29)	0.35 (0.21 to 0.6)	0.16 (0.13 to 0.19)	0.12 (0 to 0.24)	0.11 (-0.03 to 0.26)
Kiribati	248.57 (205.32 to 296.57)	62.7 (30.91 to 111.04)	0.83 (0.31 to 1.65)	-0.1 (-0.13 to -0.07)	0.49 (0.44 to 0.55)	0.69 (0.61 to 0.77)
Kuwait	270.16 (210.55 to 341.38)	27.9 (19.78 to 39.28)	0.19 (0.15 to 0.24)	-0.41 (-0.68 to -0.14)	-2.33 (-3.27 to -1.38)	-4.47 (-5.72 to -3.2)
Kyrgyzstan	198.28 (155.63 to 248.87)	38.6 (30.27 to 48.71)	0.45 (0.35 to 0.56)	1.07 (0.99 to 1.15)	0 (-0.41 to 0.4)	-0.4 (-0.99 to 0.19)
Lao People's Democratic Republic	286.04 (228.6 to 352.29)	77.54 (46.86 to 123.08)	1.05 (0.53 to 1.85)	-0.23 (-0.43 to -0.03)	-1.09 (-1.18 to -1.01)	-1.41 (-1.54 to -1.29)
Latvia	240.71 (188.47 to 307.03)	66.57 (53.23 to 82.72)	0.85 (0.68 to 1.05)	1.42 (1.38 to 1.46)	-0.53 (-1.35 to 0.29)	-1.13 (-2.14 to -0.11)
Lebanon	258.35 (204.95 to 318.47)	46.71 (29.15 to 67.93)	0.51 (0.25 to 0.84)	-0.07 (-0.24 to 0.11)	-2.39 (-2.5 to -2.28)	-3.3 (-3.47 to -3.12)
Lesotho	258.47 (193.1 to 338.67)	44.4 (24.4 to 75.5)	0.46 (0.17 to 0.95)	0.14 (0.02 to 0.27)	1.71 (1.06 to 2.38)	3.32 (1.86 to 4.8)
Liberia	180.44 (141.63 to 226.38)	36.19 (20.98 to 63.1)	0.43 (0.19 to 0.88)	0.05 (-0.04 to 0.13)	-0.31 (-0.57 to -0.05)	-0.57 (-0.95 to -0.19)
Libya	247.61 (198.75 to 307.32)	52.66 (31.78 to 79.14)	0.66 (0.31 to 1.12)	0.29 (0.07 to 0.5)	1.35 (1.04 to 1.66)	1.88 (1.43 to 2.33)
Lithuania	249.53 (196.14 to 317.04)	56.66 (45.17 to 70.51)	0.71 (0.56 to 0.86)	1.46 (1.34 to 1.58)	0.35 (-0.44 to 1.15)	0.03 (-1.08 to 1.14)
Luxembourg	481.36 (366.97 to 618.32)	33.33 (21.24 to 50.47)	0.09 (0.07 to 0.11)	1.41 (1.35 to 1.46)	-0.59 (-0.72 to -0.45)	-4.81 (-5.33 to -4.29)
Madagascar	169.02 (140.06 to 204.73)	50.28 (29.89 to 81.2)	0.66 (0.32 to 1.17)	-0.15 (-0.19 to -0.11)	-0.36 (-0.42 to -0.3)	-0.51 (-0.59 to -0.43)
Malawi	179.46 (147.25 to 218.53)	40.77 (24 to 65.39)	0.48 (0.22 to 0.9)	-0.28 (-0.33 to -0.22)	-0.72 (-0.79 to -0.65)	-1 (-1.07 to -0.92)
Malaysia	259.62 (197.83 to 329.39)	41.69 (27.46 to 59.98)	0.47 (0.26 to 0.77)	-0.15 (-0.32 to 0.01)	-0.9 (-1.03 to -0.77)	-1.14 (-1.36 to -0.91)
Maldives	233.99 (177.59 to 299.25)	31.84 (20.07 to 48.06)	0.3 (0.15 to 0.54)	-0.51 (-0.71 to -0.3)	-3.45 (-3.54 to -3.37)	-5.03 (-5.17 to -4.89)
Mali	168.63 (129.01 to 214.14)	39.94 (23.34 to 68.49)	0.51 (0.24 to 1)	-0.21 (-0.28 to -0.14)	-1.15 (-1.25 to -1.05)	-1.5 (-1.64 to -1.36)
Malta	973.79 (838.55 to 1102.96)	68.31 (46.07 to 94.97)	0.2 (0.16 to 0.24)	1.68 (1.64 to 1.72)	0.27 (0.13 to 0.41)	-3.08 (-3.53 to -2.62)
Marshall Islands	261.39 (213.49 to 316.36)	82.33 (37.87 to 162.61)	1.16 (0.4 to 2.55)	-0.04 (-0.1 to 0.03)	1.41 (1.35 to 1.48)	1.75 (1.67 to 1.83)
Mauritania	185.21 (147.25 to 230.63)	26.71 (15.69 to 47.45)	0.26 (0.11 to 0.61)	-0.01 (-0.04 to 0.03)	-1.17 (-1.29 to -1.05)	-1.66 (-1.97 to -1.36)
Mauritius	401.38 (320.4 to 490.34)	147.44 (117.11 to 181.56)	2.19 (1.68 to 2.73)	-0.16 (-0.32 to 0)	-0.17 (-0.53 to 0.19)	-0.26 (-0.71 to 0.19)
Mexico	223.78 (164.37 to 296.92)	69.24 (57.75 to 82.32)	0.94 (0.77 to 1.1)	-0.69 (-0.78 to -0.59)	1.18 (0.99 to 1.36)	1.71 (1.4 to 2.03)
Micronesia (Federated States of)	241.12 (194 to 292.41)	46.01 (26.5 to 77.53)	0.55 (0.25 to 1.1)	-0.01 (-0.06 to 0.05)	0.17 (0.1 to 0.25)	0.17 (0.05 to 0.29)
Monaco	527.36 (406.2 to 677.28)	35.11 (22.03 to 53.75)	0.07 (0.04 to 0.12)	1.44 (1.38 to 1.49)	1.15 (1.05 to 1.24)	-0.91 (-1.04 to -0.79)
Mongolia	188.46 (148.77 to 233.76)	41.99 (29.34 to 56.42)	0.53 (0.34 to 0.76)	1.05 (1.02 to 1.08)	-0.94 (-1.96 to 0.1)	-1.51 (-2.28 to -0.73)
Montenegro	439.9 (361.71 to 533.77)	66.11 (48.21 to 89.29)	0.67 (0.44 to 0.95)	1.09 (1.03 to 1.16)	0.13 (-0.25 to 0.52)	-0.39 (-0.87 to 0.09)
Morocco	299.1 (235.59 to 368.56)	49.37 (31.05 to 75.39)	0.55 (0.27 to 0.98)	0.39 (0.34 to 0.44)	0.25 (0.13 to 0.37)	0.27 (0.12 to 0.42)
Mozambique	204.52 (169.64 to 246.13)	48.78 (28.7 to 79.23)	0.6 (0.28 to 1.11)	-0.01 (-0.07 to 0.05)	-0.09 (-0.24 to 0.05)	-0.12 (-0.47 to 0.23)
Myanmar	357.16 (279.01 to 450.28)	113.38 (68.95 to 180.39)	1.66 (0.86 to 2.89)	-0.37 (-0.6 to -0.14)	-2.29 (-2.56 to -2.03)	-2.67 (-3.02 to -2.31)
Namibia	211.76 (160.88 to 271.81)	29.39 (16.87 to 48.3)	0.25 (0.1 to 0.54)	-0.07 (-0.14 to 0)	-0.71 (-0.82 to -0.6)	-1.28 (-1.49 to -1.06)
Nauru	211.99 (175.69 to 251.53)	49.89 (25.3 to 95.12)	0.65 (0.25 to 1.42)	-0.21 (-0.31 to -0.11)	0.16 (0.04 to 0.28)	0.24 (0.07 to 0.4)
Nepal	167.97 (133.23 to 210.89)	53.6 (32.55 to 84.39)	0.79 (0.42 to 1.36)	0.31 (0.25 to 0.36)	-0.76 (-0.88 to -0.64)	-0.91 (-1.05 to -0.77)
Netherlands	583.06 (452.31 to 719.74)	44.89 (30.2 to 64.89)	0.17 (0.14 to 0.2)	1.39 (1.27 to 1.51)	-0.43 (-0.52 to -0.35)	-3.91 (-4.33 to -3.48)
New Zealand	358.06 (277.31 to 461.14)	28.08 (18.93 to 40.69)	0.11 (0.09 to 0.13)	-0.02 (-0.11 to 0.07)	-2.04 (-2.45 to -1.64)	-5.73 (-6.88 to -4.56)
Nicaragua	131.96 (106.65 to 159.53)	30 (19.29 to 44.71)	0.36 (0.2 to 0.59)	0.08 (-0.02 to 0.18)	-0.97 (-1.45 to -0.49)	-1.36 (-1.99 to -0.72)
Niger	166.76 (127.36 to 214.58)	28.06 (16.25 to 45.98)	0.3 (0.13 to 0.6)	-0.06 (-0.15 to 0.03)	-0.92 (-1.03 to -0.81)	-1.46 (-1.67 to -1.26)
Nigeria	176.04 (131.09 to 236.78)	24.84 (16.35 to 35.99)	0.24 (0.13 to 0.41)	0.09 (0.04 to 0.14)	-1.43 (-1.53 to -1.32)	-2.35 (-2.54 to -2.17)
Niue	187.22 (149.81 to 228.41)	48.04 (27.07 to 77.81)	0.61 (0.29 to 1.1)	-0.04 (-0.1 to 0.01)	1.48 (1.03 to 1.93)	1.91 (1.22 to 2.6)
North Macedonia	414.3 (318 to 530.57)	70.35 (49.13 to 96.1)	0.77 (0.46 to 1.15)	0.87 (0.83 to 0.91)	-1.33 (-1.81 to -0.85)	-2.09 (-2.6 to -1.57)
Northern Mariana Islands	131.6 (102.83 to 164.23)	10.95 (6.5 to 16.92)	0.05 (0.02 to 0.09)	0.03 (-0.01 to 0.08)	-0.38 (-0.62 to -0.14)	-1.34 (-1.99 to -0.69)
Norway	918.1 (729.54 to 1132.11)	64.15 (44.27 to 91.78)	0.17 (0.16 to 0.18)	1.09 (1.07 to 1.11)	-0.19 (-0.32 to -0.05)	-3.65 (-3.98 to -3.31)

Oman	750.25 (599.16 to 923.44)	92.11 (64.88 to 126)	0.79 (0.48 to 1.21)	0.01 (-0.07 to 0.09)	-1.3 (-1.55 to -1.04)	-1.93 (-2.25 to -1.61)
Pakistan	332.46 (248.59 to 442.62)	109.66 (71.14 to 159.11)	1.56 (0.93 to 2.41)	0.38 (0.34 to 0.42)	0.79 (0.66 to 0.92)	0.84 (0.68 to 1)
Palau	203.27 (162.79 to 247.11)	41.02 (24.48 to 63.78)	0.46 (0.23 to 0.81)	-0.06 (-0.09 to -0.03)	1.04 (0.91 to 1.16)	1.39 (1.25 to 1.53)
Palestine	343.67 (252.69 to 446.72)	31.84 (20.4 to 46.57)	0.17 (0.09 to 0.28)	0.4 (0.32 to 0.49)	-0.41 (-0.52 to -0.31)	-1.56 (-1.72 to -1.41)
Panama	181.59 (139.04 to 227.75)	34.12 (25.31 to 44.94)	0.38 (0.27 to 0.5)	0.44 (0.4 to 0.49)	0.16 (-0.18 to 0.49)	0.09 (-0.39 to 0.58)
Papua New Guinea	170.57 (138.96 to 206.88)	49.07 (26.95 to 83.97)	0.67 (0.3 to 1.26)	0.15 (0.13 to 0.17)	0.31 (0.23 to 0.4)	0.32 (0.22 to 0.42)
Paraguay	262.11 (205.87 to 327.23)	38.78 (24.4 to 56.28)	0.36 (0.17 to 0.6)	0.64 (0.61 to 0.68)	0.43 (0.29 to 0.57)	0.27 (0.04 to 0.5)
Peru	121.62 (96.32 to 152.57)	16.52 (9.89 to 25.43)	0.15 (0.07 to 0.28)	0.52 (0.5 to 0.55)	-0.23 (-0.54 to 0.08)	-1.03 (-1.25 to -0.81)
Philippines	292.39 (215.72 to 391.91)	59.2 (43.06 to 78.72)	0.72 (0.48 to 1.01)	-0.41 (-0.49 to -0.32)	-0.26 (-0.41 to -0.11)	-0.18 (-0.37 to 0.02)
Poland	279.23 (209.28 to 368.19)	30.16 (22.52 to 40.93)	0.21 (0.18 to 0.25)	0.78 (0.67 to 0.88)	-2.28 (-2.49 to -2.07)	-4.44 (-4.82 to -4.06)
Portugal	481.35 (373.13 to 610.08)	34.29 (22.38 to 50.85)	0.1 (0.08 to 0.13)	1.61 (1.52 to 1.69)	-0.26 (-0.42 to -0.1)	-4.23 (-4.79 to -3.66)
Puerto Rico	469.87 (369.97 to 581.46)	103.03 (80.55 to 132.3)	1.26 (0.95 to 1.63)	-0.47 (-0.63 to -0.31)	-1.37 (-1.72 to -1.03)	-1.69 (-2.18 to -1.2)
Qatar	406.9 (302.84 to 533.73)	36.24 (22.72 to 54.37)	0.18 (0.08 to 0.33)	0.51 (0.44 to 0.59)	-1.03 (-1.25 to -0.81)	-2.98 (-3.41 to -2.56)
Republic of Korea	332.84 (260.24 to 422.18)	26.72 (16.95 to 40.03)	0.12 (0.07 to 0.2)	0.27 (0.16 to 0.37)	-2.25 (-2.38 to -2.13)	-5.08 (-5.36 to -4.8)
Republic of Moldova	214.29 (167.67 to 275.87)	64.19 (52.07 to 79.3)	0.84 (0.68 to 1.01)	1.19 (1.14 to 1.25)	-0.03 (-1.05 to 0.99)	-0.47 (-1.78 to 0.86)
Romania	206.16 (163.8 to 259.01)	23.3 (16.55 to 32.13)	0.17 (0.14 to 0.22)	1.26 (1.24 to 1.29)	-2.27 (-2.55 to -1.98)	-4.43 (-4.83 to -4.02)
Russian Federation	338.88 (264.2 to 431.13)	57.63 (48.86 to 69.9)	0.61 (0.54 to 0.68)	2.06 (1.86 to 2.26)	-0.13 (-1.59 to 1.35)	-0.99 (-2.52 to 0.56)
Rwanda	197.64 (163.53 to 237.34)	42.04 (22.31 to 74.3)	0.48 (0.18 to 1.02)	-0.27 (-0.37 to -0.17)	-2.48 (-2.93 to -2.03)	-3.36 (-4.01 to -2.71)
Saint Kitts and Nevis	349.08 (277.39 to 433.68)	55.89 (40.73 to 74.51)	0.54 (0.36 to 0.75)	-1.13 (-1.29 to -0.97)	-3.87 (-4.06 to -3.69)	-5.3 (-5.63 to -4.97)
Saint Lucia	290.66 (235.5 to 353.97)	80.62 (61.67 to 103.26)	1.07 (0.78 to 1.41)	-0.91 (-1.07 to -0.75)	-2.39 (-2.7 to -2.07)	-2.89 (-3.28 to -2.5)
Saint Vincent and the Grenadines	404.43 (331.31 to 490.43)	127.63 (98.2 to 162.13)	1.77 (1.3 to 2.33)	-0.91 (-1.07 to -0.76)	-1.6 (-2.15 to -1.05)	-1.79 (-2.5 to -1.08)
Samoa	175.56 (140.92 to 212.16)	33.02 (20.11 to 52.7)	0.39 (0.19 to 0.72)	0.01 (-0.02 to 0.04)	0.53 (0.41 to 0.64)	0.79 (0.61 to 0.97)
San Marino	544.71 (418.96 to 697.31)	36.41 (22.24 to 55.69)	0.07 (0.04 to 0.12)	1.63 (1.57 to 1.69)	0.99 (0.89 to 1.09)	-2.41 (-3.03 to -1.78)
Sao Tome and Principe	199.09 (154.63 to 249.71)	32.35 (19.64 to 50.59)	0.35 (0.16 to 0.64)	0.16 (0.13 to 0.19)	-0.04 (-0.37 to 0.3)	-0.16 (-0.6 to 0.28)
Saudi Arabia	516.66 (384.27 to 972.88)	52.04 (30.44 to 85.45)	0.39 (0.15 to 0.77)	1.16 (1.1 to 1.22)	0.18 (-0.03 to 0.4)	-0.54 (-0.73 to -0.35)
Senegal	192.24 (150.18 to 242.91)	36.7 (21.74 to 60.39)	0.43 (0.2 to 0.84)	0.01 (-0.07 to 0.09)	-0.67 (-0.96 to -0.38)	-1 (-1.41 to -0.58)
Serbia	366.11 (293.58 to 451.87)	50.92 (36.19 to 68.8)	0.48 (0.32 to 0.7)	0.92 (0.81 to 1.04)	-1.68 (-2.1 to -1.27)	-2.85 (-3.23 to -2.46)
Seychelles	233.04 (181.46 to 294.34)	44.14 (27.35 to 64.05)	0.52 (0.27 to 0.83)	-0.14 (-0.21 to -0.07)	-0.35 (-0.57 to -0.13)	-0.51 (-0.82 to -0.2)
Sierra Leone	185.73 (145.89 to 235.85)	32.7 (19.73 to 53.66)	0.36 (0.18 to 0.71)	0.13 (0.1 to 0.16)	0.26 (-0.08 to 0.6)	0.3 (-0.17 to 0.77)
Singapore	268.85 (209.96 to 340.59)	17.31 (10.19 to 27.06)	0.02 (0.02 to 0.03)	0.36 (0.3 to 0.42)	-1.67 (-1.82 to -1.52)	-7.45 (-7.8 to -7.1)
Slovakia	258.26 (201.07 to 324.29)	33.52 (23.27 to 46.3)	0.29 (0.19 to 0.43)	1.17 (1.08 to 1.27)	-0.69 (-0.89 to -0.48)	-2.11 (-2.53 to -1.68)
Slovenia	264.72 (205.21 to 338.97)	25.55 (17.35 to 37.21)	0.14 (0.11 to 0.18)	1.09 (0.86 to 1.31)	-2.03 (-2.29 to -1.77)	-4.89 (-5.31 to -4.48)
Solomon Islands	270.66 (218.2 to 327.56)	64.97 (38.33 to 110.19)	0.86 (0.43 to 1.64)	0.32 (0.24 to 0.4)	0.8 (0.62 to 0.99)	0.99 (0.81 to 1.16)
Somalia	215.38 (179.47 to 258.93)	63.45 (36.16 to 106.97)	0.85 (0.39 to 1.62)	-0.12 (-0.18 to -0.06)	0.07 (-0.01 to 0.15)	0.02 (-0.08 to 0.12)
South Africa	225.08 (168.46 to 302.82)	36.39 (25.56 to 49.91)	0.38 (0.24 to 0.55)	-0.03 (-0.07 to 0.02)	-0.62 (-1.55 to 0.32)	-0.89 (-2.28 to 0.52)
South Sudan	169.4 (140.04 to 205.32)	47.85 (26.76 to 83.47)	0.62 (0.28 to 1.22)	-0.11 (-0.15 to -0.06)	0.07 (-0.12 to 0.27)	0.03 (-0.25 to 0.31)
Spain	681.83 (582.51 to 798.25)	46.46 (30.31 to 68.48)	0.06 (0.05 to 0.07)	2.02 (1.9 to 2.14)	0.73 (0.5 to 0.95)	-4.84 (-5.16 to -4.51)
Sri Lanka	274.91 (212.94 to 351.55)	53.37 (33.24 to 80.64)	0.62 (0.29 to 1.06)	0.26 (0.23 to 0.3)	-0.5 (-0.59 to -0.4)	-0.97 (-1.2 to -0.74)
Sudan	222.48 (174.4 to 277.19)	45.38 (25.34 to 75.53)	0.57 (0.24 to 1.07)	0.29 (0.23 to 0.35)	-0.03 (-0.2 to 0.13)	-0.24 (-0.45 to -0.04)
Suriname	221.07 (177.66 to 267.73)	65.17 (40.07 to 99.25)	0.88 (0.46 to 1.45)	-0.44 (-0.52 to -0.35)	-0.81 (-1.22 to -0.4)	-0.84 (-1.35 to -0.32)
Sweden	610.95 (472.8 to 778.23)	46.41 (32.35 to 66.16)	0.22 (0.18 to 0.27)	0.53 (0.5 to 0.56)	-0.79 (-1 to -0.58)	-2.62 (-2.79 to -2.46)
Switzerland	491.26 (384.15 to 628.48)	33.07 (20.77 to 50.53)	0.07 (0.06 to 0.09)	1.29 (1.2 to 1.37)	-0.57 (-0.79 to -0.36)	-5.42 (-5.61 to -5.23)
Syrian Arab Republic	361.76 (278.82 to 455.6)	40.43 (26.53 to 58.43)	0.3 (0.16 to 0.52)	0.08 (0.01 to 0.15)	-1 (-1.11 to -0.89)	-2.03 (-2.28 to -1.78)
Taiwan (Province of China)	224.72 (183.52 to 282.84)	49.93 (40.41 to 61.42)	0.63 (0.52 to 0.76)	0.03 (-0.13 to 0.18)	-1.56 (-2.1 to -1.02)	-2.26 (-2.93 to -1.59)
Tajikistan	256.68 (204.4 to 315.07)	60.02 (38.51 to 86.53)	0.75 (0.42 to 1.18)	0.84 (0.8 to 0.88)	-0.55 (-1.41 to 0.31)	-0.89 (-1.79 to 0.02)
Thailand	200.89 (157.94 to 248.38)	42.7 (27.71 to 64.01)	0.54 (0.3 to 0.9)	-0.46 (-0.86 to -0.05)	-1.05 (-1.56 to -0.54)	-1.43 (-2.15 to -0.71)

Timor-Leste	191.96 (148.98 to 240.47)	45.25 (27.22 to 71.06)	0.58 (0.28 to 1.03)	0.04 (-0.04 to 0.13)	-0.16 (-0.56 to 0.24)	-0.3 (-0.85 to 0.24)
Togo	178.43 (141.53 to 224.27)	31.78 (18.64 to 52.47)	0.35 (0.16 to 0.69)	-0.07 (-0.11 to -0.03)	-0.68 (-0.86 to -0.51)	-1.03 (-1.28 to -0.78)
Tokelau	203.69 (161.24 to 246.7)	46.36 (28.49 to 71.08)	0.57 (0.3 to 0.97)	0.01 (-0.07 to 0.08)	0.72 (0.28 to 1.15)	0.81 (0.17 to 1.46)
Tonga	172.94 (136.45 to 212.84)	33.96 (20.17 to 54.4)	0.41 (0.2 to 0.76)	-0.08 (-0.11 to -0.04)	-0.17 (-0.22 to -0.12)	-0.18 (-0.24 to -0.12)
Trinidad and Tobago	296.25 (238.72 to 355.15)	110.36 (80.34 to 148.98)	1.61 (1.11 to 2.26)	-0.75 (-1 to -0.5)	-1.28 (-1.9 to -0.65)	-1.57 (-2.18 to -0.95)
Tunisia	235.47 (185.86 to 294.99)	30.44 (19.74 to 44.48)	0.28 (0.14 to 0.47)	0.13 (0.07 to 0.19)	0.04 (-0.08 to 0.15)	-0.16 (-0.3 to -0.01)
Turkey	280.26 (214.65 to 354.23)	29.8 (19.67 to 43.33)	0.18 (0.1 to 0.28)	0.12 (0.02 to 0.22)	-1.65 (-1.83 to -1.47)	-3.64 (-3.96 to -3.32)
Turkmenistan	327.77 (271.26 to 399.67)	111.99 (83.03 to 149.32)	1.55 (1.1 to 2.16)	1.2 (1.16 to 1.23)	1.32 (0.65 to 2)	1.25 (0.49 to 2.02)
Tuvalu	225.02 (178.93 to 275.12)	42.13 (25.47 to 67.81)	0.5 (0.24 to 0.93)	-0.16 (-0.19 to -0.12)	-0.44 (-0.57 to -0.31)	-0.6 (-0.85 to -0.36)
Uganda	172.55 (141.85 to 208.76)	39.8 (21.89 to 67.25)	0.47 (0.19 to 0.93)	0.07 (0.02 to 0.12)	0.29 (-0.04 to 0.62)	0.28 (-0.22 to 0.78)
Ukraine	290.76 (216.43 to 387.43)	53.73 (35.79 to 76.71)	0.6 (0.33 to 0.92)	1.09 (1.05 to 1.13)	-1.63 (-2.45 to -0.79)	-2.48 (-3.39 to -1.56)
United Arab Emirates	391.12 (294.73 to 513.59)	37.28 (24.11 to 54.94)	0.23 (0.13 to 0.37)	0.38 (0.29 to 0.47)	-0.17 (-0.5 to 0.17)	-1.18 (-1.59 to -0.78)
United Kingdom	401.95 (309.02 to 523.67)	32.71 (23.98 to 45.63)	0.18 (0.17 to 0.19)	0.15 (-0.1 to 0.4)	-0.71 (-0.91 to -0.51)	-2.21 (-2.56 to -1.86)
United Republic of Tanzania	231.46 (170.83 to 297.67)	42.08 (24.25 to 66.46)	0.44 (0.2 to 0.82)	-0.01 (-0.11 to 0.08)	-0.41 (-0.5 to -0.33)	-0.53 (-0.72 to -0.33)
United States of America	550.23 (446.42 to 680.54)	73.12 (61.5 to 88.98)	0.75 (0.71 to 0.79)	0.32 (0.26 to 0.37)	0.09 (-0.14 to 0.32)	-1.13 (-1.38 to -0.87)
United States Virgin Islands	326.23 (255.62 to 398.75)	85.86 (47.49 to 134.93)	1.13 (0.48 to 1.97)	-0.51 (-0.61 to -0.41)	-0.93 (-1.12 to -0.74)	-0.09 (-0.46 to 0.29)
Uruguay	358.01 (283.43 to 445.9)	32.39 (23.08 to 45.05)	0.2 (0.15 to 0.24)	0.51 (0.49 to 0.53)	-0.32 (-0.49 to -0.15)	-1.48 (-1.84 to -1.11)
Uzbekistan	452.38 (371.15 to 546.21)	97.07 (74.45 to 124.13)	1.17 (0.87 to 1.54)	1.33 (1.29 to 1.37)	1.13 (0.61 to 1.65)	1.07 (0.45 to 1.69)
Vanuatu	195.48 (155.14 to 236.8)	39.42 (23.49 to 63.92)	0.49 (0.23 to 0.91)	0.07 (0.01 to 0.12)	0.75 (0.54 to 0.95)	1.02 (0.73 to 1.3)
Venezuela (Bolivarian Republic of)	124.31 (101.27 to 151.15)	50.28 (36.52 to 67.33)	0.71 (0.49 to 0.98)	-0.07 (-0.13 to -0.01)	0.8 (0.2 to 1.41)	0.93 (0.2 to 1.66)
Viet Nam	251.55 (194.01 to 317.61)	45.58 (27.76 to 69.69)	0.53 (0.24 to 0.95)	0.17 (0.13 to 0.22)	-0.97 (-1.04 to -0.91)	-1.45 (-1.53 to -1.36)
Yemen	215.89 (168.98 to 267.35)	34.39 (19.07 to 61.81)	0.38 (0.14 to 0.87)	0.32 (0.25 to 0.4)	-0.01 (-0.29 to 0.26)	-0.34 (-0.72 to 0.05)
Zambia	187.71 (155.47 to 226.86)	44.73 (25.97 to 73.45)	0.54 (0.25 to 1.03)	-0.34 (-0.42 to -0.26)	-1.23 (-1.39 to -1.07)	-1.62 (-1.82 to -1.42)
Zimbabwe	149.35 (115.7 to 189.78)	30.66 (16.91 to 50.65)	0.35 (0.14 to 0.66)	0.27 (0.16 to 0.38)	2.44 (1.66 to 3.22)	4.11 (2.58 to 5.66)

ASMR = age standardized deaths rate; T1DM = T1DM=type 1 diabetes mellitus; WCBA = women of childbearing age; AAPC = average annual percentage change; SDI = socio-demographic index; 95% UI = 95% uncertainty interval; 95% CI = 95% confidence interval.
